# Continuous multi-omics pathway enrichment analysis resolves hidden functional heterogeneity

**DOI:** 10.1093/bib/bbag328

**Published:** 2026-06-29

**Authors:** Sareh Amerifar, Andreas Kopf, Steffen Sass, Zahra Moslehi, Dennis Hecker, Julius C Enssle, Marcel H Schulz, Thomas Oellerich, Fabian J Theis, Florian Buettner

**Affiliations:** MedicineII-Hematology and Oncology, University Hospital Frankfurt, Theodor-Stern-Kai 7, 60590 Frankfurt am Main, Hessen, Germany; Goethe University Frankfurt, Theodor-Stern-Kai 7, 60590 Frankfurt am Main, Hessen, Germany; German Cancer Consortium (DKTK), partner site Frankfurt/Mainz, a partnership between DKFZ and UCT Frankfurt-Marburg, Theodor-Stern-Kai 7, 60590 Frankfurt am Main, Hessen, Germany; German Cancer Consortium (DKTK), partner site Frankfurt/Mainz, a partnership between DKFZ and UCT Frankfurt-Marburg, Theodor-Stern-Kai 7, 60590 Frankfurt am Main, Hessen, Germany; German Cancer Research Center (DKFZ), Im Neuenheimer Feld 280, 69120 Heidelberg, Baden-Württemberg, Germany; Digitec Galaxus AG, Pfingstweidstrasse 60b, 8005 Zürich, Switzerland; Institute of Computational Biology, Helmholtz Munich, Ingolstädter Landstraße 1, 85764 Neuherberg, Bavaria, Germany; Pharma Technical Development, Roche Diagnostics GmbH, Nonnenwald 2, 82377 Penzberg, Bavaria, Germany; MedicineII-Hematology and Oncology, University Hospital Frankfurt, Theodor-Stern-Kai 7, 60590 Frankfurt am Main, Hessen, Germany; German Cancer Consortium (DKTK), partner site Frankfurt/Mainz, a partnership between DKFZ and UCT Frankfurt-Marburg, Theodor-Stern-Kai 7, 60590 Frankfurt am Main, Hessen, Germany; Institute for Computational Genomic Medicine, Goethe University Frankfurt, Theodor-Stern-Kai 7, 60590 Frankfurt am Main, Hessen, Germany; MedicineII-Hematology and Oncology, University Hospital Frankfurt, Theodor-Stern-Kai 7, 60590 Frankfurt am Main, Hessen, Germany; German Cancer Consortium (DKTK), partner site Frankfurt/Mainz, a partnership between DKFZ and UCT Frankfurt-Marburg, Theodor-Stern-Kai 7, 60590 Frankfurt am Main, Hessen, Germany; Frankfurt Cancer Institute (FCI), Georg-Speyer-Haus, Paul-Ehrlich-Straße 42-44, 60596 Frankfurt am Main, Hessen, Germany; Lymphoid Malignancies Branch, National Cancer Institute, National Institutes of Health, 9000 Rockville Pike, Bethesda, MD 20892, United States; Institute for Computational Genomic Medicine, Goethe University Frankfurt, Theodor-Stern-Kai 7, 60590 Frankfurt am Main, Hessen, Germany; MedicineII-Hematology and Oncology, University Hospital Frankfurt, Theodor-Stern-Kai 7, 60590 Frankfurt am Main, Hessen, Germany; Goethe University Frankfurt, Theodor-Stern-Kai 7, 60590 Frankfurt am Main, Hessen, Germany; German Cancer Consortium (DKTK), partner site Frankfurt/Mainz, a partnership between DKFZ and UCT Frankfurt-Marburg, Theodor-Stern-Kai 7, 60590 Frankfurt am Main, Hessen, Germany; German Cancer Research Center (DKFZ), Im Neuenheimer Feld 280, 69120 Heidelberg, Baden-Württemberg, Germany; Frankfurt Cancer Institute (FCI), Georg-Speyer-Haus, Paul-Ehrlich-Straße 42-44, 60596 Frankfurt am Main, Hessen, Germany; Institute of Computational Biology, Helmholtz Munich, Ingolstädter Landstraße 1, 85764 Neuherberg, Bavaria, Germany; School of Life Sciences, Technical University of Munich, Alte Akademie 8, 85354 Freising, Bavaria, Germany; School of Computation, Information and Technology, Technical University of Munich, Arcisstraße 21, 80333 Munich, Bavaria, Germany; Goethe University Frankfurt, Theodor-Stern-Kai 7, 60590 Frankfurt am Main, Hessen, Germany; German Cancer Consortium (DKTK), partner site Frankfurt/Mainz, a partnership between DKFZ and UCT Frankfurt-Marburg, Theodor-Stern-Kai 7, 60590 Frankfurt am Main, Hessen, Germany; German Cancer Research Center (DKFZ), Im Neuenheimer Feld 280, 69120 Heidelberg, Baden-Württemberg, Germany; Frankfurt Cancer Institute (FCI), Georg-Speyer-Haus, Paul-Ehrlich-Straße 42-44, 60596 Frankfurt am Main, Hessen, Germany

**Keywords:** pathway enrichment analysis, Bayesian modeling, multi-omics integration, bioinformatics, machine learning

## Abstract

Pathway enrichment analysis is essential for extracting biological insights from complex omics datasets, yet existing methods suffer from critical limitations: excessive false discoveries, arbitrary significance thresholds, poor handling of multi-omics data, and inability to model gene dependencies. We present JOANA (Joint continuous multi-Omics enrichment ANAlysis), a novel Bayesian framework for pathway analysis with three key contributions. First, JOANA enables high specificity through continuous probabilistic modeling of significance scores using Beta mixture distributions, eliminating arbitrary thresholds while maintaining sensitivity. Second, JOANA’s multi-omics integration via Bayesian networks inherently accounts for missing values and reveals pathways invisible to single-layer analyses. Finally, we demonstrate high versatility across diverse experimental paradigms—from proteomics and transcriptomics to single-cell transcriptomics, mutation analysis, and transcriptomics–epigenomics data. In systematic comparisons on synthetic data as well as diverse real-world multi-modal datasets, JOANA achieves up to $\sim $20-fold reduction in reported pathways compared with existing methods while maintaining sensitivity for true biological signals. We implement JOANA in an open-source Python package, joanapy.

## Introduction

Pathway enrichment analysis (PEA) is a robust strategy for simplifying high-throughput data by reducing complexity while maintaining biological relevance [1]. In the context of health and disease, PEA can play an important role for identifying the relevant biological processes involved. This in turn can aid in the understanding of disease mechanisms, along with its resistance to treatment [2], and finding potential drug targets [3].

PEA is usually carried out as a subsequent step following a differential analysis (DA). It leverages the outcomes of DA, incorporating statistical metrics (e.g. test statistics, adjusted $P$-values, and/or fold changes) to elucidate and consolidate these findings via predefined biological pathways.

Within the field of PEA, numerous computational tools have been developed, each providing distinct perspectives on the complex interactions of biological pathways. The two primary categories of pathway enrichment methods are over-representation analysis (ORA) and functional class scoring (FCS).

ORA is based on statistical methods that assess whether a specific biological pathway is over-represented among significant features (e.g. genes or proteins) compared with what would be expected by chance [4–7]. The primary constraints of ORA stem from its reliance on specific thresholds on the significance score (e.g. $P$-value or adjusted $P$-value) of differentially analysis, which determines feature categorization as significant or insignificant. Standard ORA approaches then treat all significant values equally (e.g. based on Fisher’s exact test), overlooking potential dependencies among genes and pathways and neglecting the individual significance level of each feature [8]. FCS takes a different approach to overcome ORA limitations. Instead of relying on arbitrary fixed thresholds for significance scores resulting from a DA or treating all significant and insignificant values equally, FCS methods assign scores to individual genes based on quantities such as their fold change, test statistic, and/or statistical significance score in the analysis. These scores are then combined to calculate an overall pathway score, which is assessed for significance using permutation tests or similar statistical techniques. The result is a prioritized list of pathways based on their scores [9–12]. However, FCS-based methods do not take into account pathway dependencies [8]. These limitations in the two main PEA approaches often lead to a specificity issue, causing many pathways to be reported as enriched and making the results difficult to interpret [1, 13]. Furthermore, most tools are limited to working with single-omics data, which becomes increasingly problematic with the emergence of multi-omics data. Recently, several tools have emerged to address this multi-omics challenge. These tools can be grouped into three primary categories: those that combine data at the level of individual genes, those that combine data at the level of biological pathways, and those that comprehensively integrate data using machine learning techniques. Combining data at the gene-level offers an advantage over pathway-level integration by considering relationships among different types of data instead of treating each type independently. However, both gene-level and pathway-level integration methods ultimately face limitations inherited from ORA and FCS due to their underlying methodologies in the pathway enrichment phase.

In addition, PEA of multi-omics data introduces an integration-related challenge, namely managing missing values, where certain entries across data modalities may lack measurements. In this work, we introduce a new algorithm for PEA of multi-modal data in the FCS paradigm. We refer to our algorithm as JOANA (Joint continuous multi-Omics enrichment ANAlysis). JOANA employs Bayesian inference techniques to determine the probability of pathway activity, drawing from the underlying Bayesian network. Unlike ORA methods, it does not treat all features the same way; instead, it evaluates their statistical significance scores (e.g. adjusted $P$-values) rather than simply categorizing them as either insignificant or significant (i.e. 0 or 1). This threshold-free modeling approach is implemented by fitting a continues distribution of the significance score resulting from a DA. Unlike existing FCS methods, JOANA explicitly models pathway dependencies. Furthermore, JOANA is capable of handling both single and multi-omics data while also accounting for dependencies between different data modalities. Finally, it can effectively manage missing data across various data modalities.

In summary, JOANA improves upon existing PEA methods by:

- Accounting for gene-specific significance levels: JOANA explicitly models the continuous distribution over significance scores from DA rather than relying on strict categorization as insignificant or significant. It thereby eliminates the dependency on arbitrarily fixed thresholds.
- Modeling gene- and pathway-dependencies: JOANA models the dependencies between genes within the same pathway and accounts for interactions and dependencies between different pathways via a Bayesian network.
- Handling multi-omics data: JOANA integrates data from multiple omics layers and naturally allows for missing values.
- Increasing specificity: Via its joint modeling paradigm, JOANA consistently reduces the number of false positives (FPs) in enriched pathways, leading to better interpretability of results.

We implemented JOANA as an open-source Python package, joanapy, accompanied by a comprehensive tutorial (https://github.com/MLO-lab/joanapy).

## Materials and methods

### Algorithm

**Figure 1.**
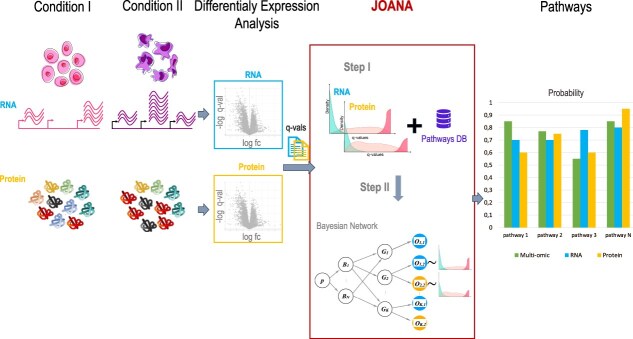
Overview of JOANA. JOANA starts by taking the results from a DA as input. In Step I, it models this input data as a mixture of Beta distributions. The input to Step II is this parameterization of the DA results alongside predefined pathways. It is then used in a Bayesian Network to calculate the probability of activity for gene sets associated with each data modality, as well as the common activity shared across all layers.

Here, we propose JOANA as a solution for uncovering the functional diversity between different biological groups or conditions. In brief, JOANA takes gene lists and their corresponding significance scores (i.e. adjusted $P$-values) obtained from a DA for each data modality as input. It then computes the probabilities of pathway activity for each data type independently and collectively across all data types (Fig. 1) via Bayesian inference. We refer to a pathway as active if it truly drives functional diversity between different biological groups. JOANA operates through two main steps that will be explained in detail in the following sections.

### Step I: Parameterizing the q-value distribution

To account for the full distribution of q-values (rather than a binarized version as in ORA models), we first parameterize it as a flexible mixture of three Beta distributions (Equation (1)). 


(1) \begin{align*} {\mathrm{BMM}} &= \mathrm{\mu_{ac}}{\mathrm{Beta}} \left( \alpha_{ac}, \beta_{ac} \right) + \mathrm{\mu_{inacI}}{\mathrm{Beta}} \left( \alpha_{\mathrm{inacI}}, \beta_{\mathrm{inacI}} \right) \notag \\ &\quad + \mathrm{\mu_{inacII}}{\mathrm{Beta}} \left( \alpha_{\mathrm{inacII}}, \beta_{\mathrm{inacII}} \right)\end{align*}

**Figure 2.**
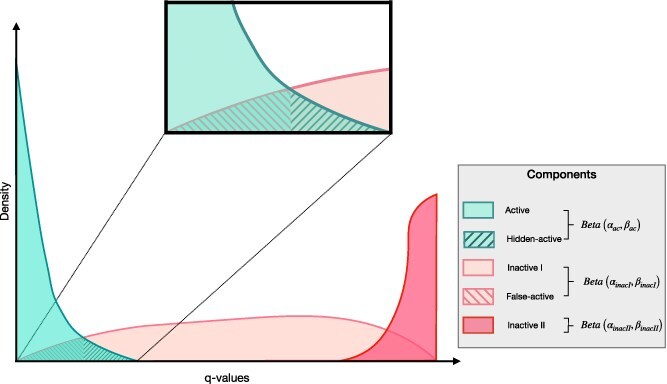
JOANA models q-values of DA using a Beta Mixture Model (BMM Equation (1)) with three components. The first component represents the active segment of the data, including any hidden active portions in the tail. The second component represents a broad distribution across the entire range of q-values (Inactive I), allowing for FPs (i.e. small q-values not related to an active pathway). Lastly, a third component models the observed data with values close to 1, with an Inactive II component; this is represented by the peak close to 1.

This Beta Mixture Model comprises three components: an active component and two inactive components (Inactive I and Inactive II, Fig. 2). We refer to them as active and inactive since, in active pathways, the q-values of all genes annotated to those pathways are drawn from the active component. Conversely, if a pathway is inactive, the q-values of genes annotated to that pathway are drawn from the inactive components.

The fundamental idea motivating these three components is based on the notion that, in scenarios where no features show significance (null hypothesis), the resulting $P$-values from DA are drawn from a Uniform distribution. However, in cases where there are truly differential features (the alternative hypothesis), the distribution of $P$-values tends to shift towards lower values, indicating stronger evidence against the null hypothesis. This tendency often results in a distinct peak of lower $P$-values. After adjusting $P$-values for multiple test using $P$-value adjustment methods (e.g. false discovery rate (FDR)) and obtaining q-values, another peak close to one is commonly observed. We model both peaks with flexible Beta distributions, parameterized with two shape parameters ($\alpha $ and $\beta $). To increase flexibility of the parametric model describing the q-value distribution and consistency with the data, we replace the Uniform distribution, with a third Beta distribution (note that a Uniform distribution is a special case with shape parameters $\alpha $ and $\beta $ equaling to 1).

To determine the parameters ($\alpha $ and $\beta $) for each component of a Beta mixture distribution, the simplest method is to optimize the log-likelihood function and find the maximum-likelihood estimation (MLE). However, Schröder and Rahmann [14] introduced an innovative hybrid parameter estimation algorithm for Beta mixture models iteratively optimizing higher-order moments. This iterated method of moments combines latent variables with the method of moments to address issues that arise with MLE, particularly when the data include boundary values (0 or 1).

In this hybrid approach, the process begins with initial guesses for each Beta distribution’s parameters in the mixture. It then iteratively computes the probabilities that each data point belongs to each component (Expectation Step). Using these probabilities, the parameters of each component are updated via weighted moments (Maximization Step). This iterative process continues until the parameters stabilize [14]. For JOANA, we implemented this approach to determine all the parameters in Equation (1).

Unlike standard ORA, which classifies data points using an arbitrarily fixed significance threshold (e.g. 0.05), JOANA was designed in the spirit of the functional scoring class, using a probabilistic approach. JOANA’s probabilistic assignment method enables the detection of hidden-active (i.e. large non-significant q-values associated with an active pathway) and false-active values (i.e. small, significant q-values not associated with any true active pathway) across all data types. Considering a significance threshold of 0.05 for q-values, hidden-active values are those with q-values >0.05, indicating they are not significant. JOANA assigns a low probability to these values being active, suggesting they might still contribute to active pathways (illustrated by the shaded green area in Fig. 2). False-active values have q-values <0.05, indicating significance. JOANA identifies these values by assigning them a low probability of being drawn from the inactive part (depicted in the shaded light pink area in Fig. 2). By allowing for both hidden-active and false-active values, JOANA is robust to observation noise in the data.

### Step II: JOANA as a Bayesian Network

**Figure 3.**
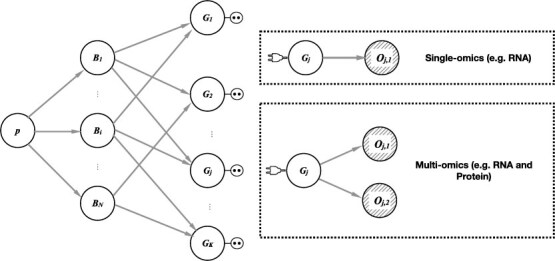
JOANA creates a Bayesian network with three layers: the biological-pathway layer, gene-response layer, and the observation layer. The network encodes pathways based on connections between ${B_{i}}$ and gene-response layer $G_{j}$. The third layer in the Bayesian network, the observation layer, allows for the execution of JOANA with two distinct model types. Firstly, the single omics model (top right panel), which only considers one input layer, such as mRNA or proteins. Secondly, JOANA can manage multi-level or multi-omics data using the multi-omics model (bottom right panel), inferring pathways that are active jointly in both views.

In this step, JOANA creates a Bayesian network (Fig. 3) which is organized in three main layers. The first layer, which we refer to as biological pathway layer, comprises nodes $B = (B_{1}, B_{2},..., B_{N})$, where each $B_{i}$ (with $i \in \{1, 2,..., N\}$) represents an individual pathway, i.e. being examined. The number of pathways tested is denoted by $N$.

The second layer is the gene-response layer, which includes nodes $G = (G_{1}, G_{2},..., G_{K})$, with $K$ representing the total number of genes of interest. Edges connecting $B_{i}$ to $G_{j}$ indicate that the gene-response node $G_{j}$ is associated with the biological pathway $B_{i}$ via a pathway database. That is, each pathway node $B_{i}$ is linked to a specific set of gene-response nodes that constitute that pathway. Connections between G and B can be represented by an adjacency matrix A, which can be generated automatically from a gmt file.

Furthermore, there is an observation layer. In the single-omics version of JOANA, this layer consists of nodes $O = (O_{1}, O_{2},..., O_{K})$, where each $O_{j}$ (with $j \in{1, 2,..., K}$) corresponds to an observed q-value of a gene (e.g. RNA expression level). Each gene-response node $G_{j}$ is connected to exactly one observation node $O_{j}$.

In the multi-omics mode of JOANA, observation nodes are denoted as $O_{j,m}$, where $m \in \{1,..,M\}$ represents the modality, and $M$ is the total number of measured modalities for gene-response $G_{j}$. Therefore, each gene-response node $G_{j}$ can have multiple observation nodes associated with it. For example, a single gene-response $G_{j}$ might be observed via q-values measured in different modalities like RNA and protein.

JOANA can function with either single-omics or multi-omics data, depending on the input it receives. For simplicity, we will first describe the model using single-omics data before specifying the details of the multi-omics implementation.

#### Single-omics modeling

In the pathway layer, the nodes $B_{i}$ are boolean random variables that follow a Bernoulli distribution $ B_{i} \sim \mathrm{Bernoulli}(p_{i}) $. Here, $p_{i}$ represents the prior probability of pathway $B_{i}$ being active (i.e. equal to 1). Random variable $p = (p_{1}, p_{2},..., p_{N})$ follows a Beta distribution parameterized by $\alpha $ and $\beta $, $ p \sim \mathrm{Beta}(\alpha , \beta )$.

Initially, the distribution of $p$ is unknown, so we introduce a prior distribution over $p$ to reflect our beliefs about this random variable. In the default configuration of JOANA, we set $\alpha = \beta = 1$ which results in an uninformative Uniform distribution of $p$. However, these values can be adjusted based on prior knowledge of the data. For example, if we anticipate that $p$ is more likely to be small, we adjust the parameters $\alpha $ and $\beta $ to bias the prior distribution towards certain values of $p$ that align with these prior beliefs (e.g. $\alpha = 1$ and $\beta = 5$).

Nodes $G_{i}$ are also boolean random variables that can take values of 1 (active) or 0 (inactive). We define the conditional probability $P(G_{j} | B)$ based on the connectivity of $G_{j}$ to biological-pathways $B$. In Equation (2), $B(G_{j})$ represents the set of pathways to which the node $G_{j}$ is associated. 


(2) \begin{align*} P({G_{j}|B}) = \begin{cases} 1 & \mathrm{if}\ \exists B_{i} \in B(G_{j}):B_{i} = 1 \\ 0 & \text otherwise \end{cases}\end{align*}

In other words, a gene-response node $G_{j}$ will be active if it is connected to at least one active pathway.

Alternatively, we can interpret Equation (2) such that a pathway will be active if all corresponding gene-response nodes connected to that pathway are active.

We utilize the probability distribution defined over the observed q-values (Equation (1)) to establish the conditional probability of $O_{j}$ given $G_{j}$: 


(3) \begin{align*}& P(O_{j}\vert G_{j}) = \begin{cases} {\mathrm{Beta}}(\alpha_{ac},\beta_{ac}), & \mathrm{if}\ G_{j}=1 \\\\ \mu_{\mathrm{inacI}} {\mathrm{Beta}}(\alpha_{\mathrm{inacI}},\beta_{\mathrm{inacI}})+ \\ \mu_{\mathrm{inacII}} {\mathrm{Beta}}(\alpha_{\mathrm{inacII}},\beta_{\mathrm{inacII}}) & \mathrm{if}\ G_{j}=0 \end{cases}\end{align*}

This means that the probability of $O_{j}$ depends on the value of $G_{j}$. If $G_{j}$ is active, the probability of $O_{j}$ is drawn from the active component of the parameterized q-value distribution. Conversely, if $G_{j}$ is inactive, the probability drawn from the inactive mixture components of the distribution.

#### Multi-omics modeling

When JOANA observes multiple data modalities, it can identify shared patterns across these layers by jointly modeling all modality-specific observations.: unlike a single-omics approach, this model includes additional observation nodes ${O_{j,m}}$ linked to $G_{j}$.

When linking all observed q-values to the gene-response $G_{j}$, we account for all modalities in the status of $G_{j}$ (whether it is active or inactive): 


(4) \begin{align*}& P(O_{j,m}|G_{j}) = \left \{ \begin{array}{@{}ll@{}} {\mathrm{Beta}}(\alpha_{\mathrm{ac,m}},\beta_{\mathrm{ac,m}}) & \mathrm{if} \ G_{j}=1 \\ \\ \mu_{\mathrm{inacI,m}} {\mathrm{Beta}}(\alpha_{\mathrm{inacI,m}},\beta_{\mathrm{inacI,m}})+ \\ \mu_{\mathrm{inacII,m}} {\mathrm{Beta}}(\alpha_{\mathrm{inacII,m}},\beta_{\mathrm{inacII,m}}) & \mathrm{if} \ G_{j}=0 \end{array}\right.\end{align*}

Note that in *step I*, all required parameters for Equations (3) and (4) ($\alpha _{c,m}, \beta _{c,m}$) where $c \in \{ac,{\mathrm{inacI,inacII}}\}$, as well as the weights ($\mu _{{\text{ inacI}},m}, \mu _{\mathrm{InacII}},m$), are initially determined using the iterated method of moments approach.

### Bayesian inference using expectation propagation

To compute the probability of pathway activity for all pathways given the observed q-values, we need to infer the marginal posterior distribution $P(B\vert O)$. As this posterior distribution is intractable, we employ expectation propagation (EP) [15] to approximate the true posterior. In brief, EP iteratively refines approximations of the posterior distributions for pathway activities by factorizing the posterior into independent factors (the latent variables) that are then iteratively updated via message passing [15]. We assessed convergence of the EP procedure by monitoring the entropy of the posterior $P(B \mid O)$ across iterations (Fig. S5). The entropy decreases rapidly and stabilizes within $\sim $20 iterations, indicating convergence. Based on this behavior, JOANA uses 30 EP iterations by default. We implemented EP via probabilistic programming using Infer.NET [16]. 


(5) \begin{align*}& \begin{aligned} P(&B,G,p\vert \mathrm{O}) = &\frac{P(p)P(B\vert p)P(G\vert B)P(O\vert G)}{P(O)} \end{aligned}\end{align*}

Algorithm 1 summarizes the full algorithm.



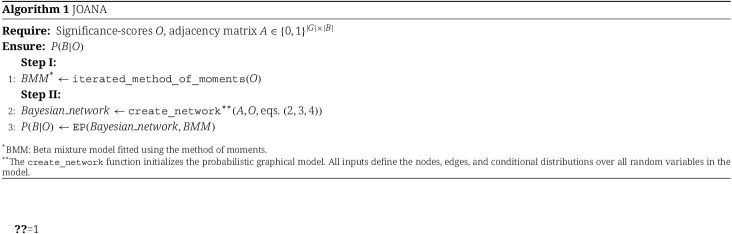



### Handling missing values

When dealing with multiple data modalities, the number of entities or identifiers may not always be consistent. For instance, when data arises from different measurement types, such as genes analyzed via single-cell RNA sequencing and proteins identified through mass spectrometry, the numbers of identifiers often differ. Consequently, JOANA’s ability to handle missing values within incomplete layers by utilizing available gene responses becomes beneficial.

Marginalization in EP handles missing values by treating them as additional latent variables and integrating over their possible values. This approach incorporates the uncertainty due to missing data directly into the inference process.

In EP, the Bayesian network’s nodes and parameters are initialized first. During the message-passing phase, the algorithm identifies observed variables with missing values. For each factor involving missing data, the expectation over the missing values is computed. This involves integrating the factor over all possible values of the missing data, weighted by their probability given the observed data and other latent variables. The updated messages, reflecting this integration, are then passed between nodes.

This process iteratively updates the beliefs (approximations of the marginal distributions) for each variable, including the missing data, until convergence is reached. The final approximations account for the uncertainty introduced by the missing data, leading to more accurate posterior distributions for the latent variables.

### Python package joanapy

JOANA is available as a Python package (joanapy). It can be configured to run either the single-omics model or the multi-omics model, which automatically infers the respective single-omics results. As input, joanapy requires a list of q-values derived from DA per data modality, and a gmt file containing biological pathways and their list of gene members.

Additionally, joanapy provides several diagnostic functions to help interpret the inferred results. These include activity barplots per data modality, pathway network plots showing the connectivity between active pathways with respect to overlapping entities, and an explicit goodness-of-fit assessment (Figs S12 and S13). The goodness-of-fit assessment compares the empirical q-value distributions to the fitted three-component Beta mixture model after moment fitting and prior to Bayesian inference. It allows users to evaluate how well the mixture assumption holds for their data and can identify potential deviations. Both visual inspection (histograms) and quantitative measures such as Kolmogorov–Smirnov (KS), and Anderson–Darling (AD) statistics are provided to assess agreement between the data and the model.

### Synthetic data

JOANA assumes that q-values from DA follow a mixture of three Beta distributions (Equation (1), Fig. 2). To avoid favoring JOANA in synthetic benchmark experiments and to explicitly evaluate its robustness under model mismatch, we generated synthetic datasets from distributions that did match the generative model underlying JOANA.

Specifically, we generated synthetic data from a diverse collection of mixture models that differ fundamentally from the three-Beta mixture used by JOANA. Across nine distinct mixture settings, we combined Beta, Exponential, Gamma, and Uniform distributions, resulting in a total of 115 200 synthetic datasets (see Supplementary Information for details). Importantly, none of these mixture settings corresponds to the three-Beta model assumed by JOANA.

**Figure 4.**
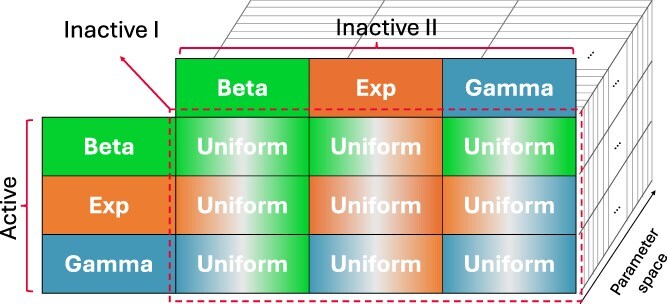
Various mixture models used for sampling and generating synthetic data. The Active component is sampled from Beta, Exponential, and Gamma distributions, and Inactive II is drawn from the same set of distributions. Inactive I is sampled from a Uniform distribution. The depth of the depicted cube represents the parameter space, formed by permutations within each distribution. This approach creates a spectrum of distributions, showcasing JOANA’s capability to handle diverse distribution shapes effectively.

We conducted sampling from various combinations involving Beta, Exponential, and Gamma distributions representing the Active and Inactive II components, together with a shared Uniform distribution representing the Inactive I component (Fig. 4). The mixture weights for the three components were also sampled. By varying distribution shapes through different parameter choices ($\alpha =(1,2,6,10)$ and $\beta =(6,11,18,25)$), we generated a broad range of distributional overlaps between active and inactive components. This design allowed us to systematically evaluate performance across varying levels of difficulty and to compare JOANA against competing PEA methods under realistic deviations from its modeling assumptions.

### Real-world data

To demonstrate JOANA’s practical usefulness in real-world applications, we evaluated it on a range of published multi-modal datasets. We first focused on two proteomics–transcriptomics studies [17, 18]. For the first study [17], DA of both transcriptomic and proteomic data was performed using the limma R package [19], following the confounding variables reported by the authors. For the second dataset [18], we directly used the significance scores provided in the original study.

To further highlight JOANA’s versatility across different data modalities, we analyzed three additional published datasets. The first dataset consists of single-cell RNA-seq data [20], for which significance scores were computed using Seurat [21]. The second dataset includes mutation profiles covering both protein-coding genes and non-coding regulatory regions; in our analysis, we focused on mutations in coding regions and promoters, using significance scores provided by the Pan-Cancer Analysis of Whole Genomes (PCAWG) Consortium [22].

The third dataset contains RNA-seq and histone ChIP-seq data derived from a high-fat and alcohol-rich diet mouse model [23]. This model is well characterized and is known to induce liver injury and metabolic dysregulation, providing a biologically grounded benchmark. Enrichment results were therefore expected to recover pathways associated with liver damage and related processes, enabling a quantitative comparison with existing multi-omics pathway enrichment methods. In the steatosis study [23], $P$-values for differential analyses between control and fatty liver mice were obtained from RNA-seq data by testing for differentially expressed genes with DESeq2 [24], and genes marked by differential chromatin domains using the SCIDDO method [25]. To attribute the results from SCIDDO to genes, we linked the regions to the nearest gene with pybedtools (v0.8.1) [26, 27]. For the location of genes, we took the most 5’ TSS from the vM21 annotation from GENCODE [28].

### Comparison to existing methods

We chose ActivePathways [29] and multiGSEA [30] as state-of-the-art tools for gene-level and pathway-level integration, respectively, to compare with JOANA. Furthermore, we included MONA [31], a machine learning-based method belonging to the ORA family, which, like JOANA, utilizes Bayesian inference for pathway enrichment, into our comparative analysis.

ActivePathway, selected to represent gene-level integration tools, utilizes Brown’s extension of the Fisher test for integrating data layers. It then sets a threshold on the results to eliminate highly insignificant integrated values before applying a ranked hypergeometric test for pathway enrichment. By employing the ranked hypergeometric test, ActivePathways aims to mitigate the issue of treating all gene values equally. However, it still fails to account for dependencies between gene values and pathways.

As a popular choice for pathway-level integration, we compared JOANA to multiGSEA, which serves as the multi-omics counterpart to GSEA, a popular and widely cited approach for single-omics data. multiGSEA conducts GSEA on each data level, thereby overcoming the single-omics limitation, and subsequently combines enriched pathway results using statistical methods. However, multiGSEA neglects dependencies between different data modalities and inherits limitations from FCS methods as discussed in the Introduction sections.

We finally compared JOANA to MONA. Similar to JOANA, MONA employs Bayesian inference for PEA. MONA is a multi-omics tool that accounts for dependencies between data layers, genes, and pathways. However, like other ORA methods, MONA has limitations in terms of defining a significance thresholds for q-values from DA. It categorizes genes simply as significant or insignificant, potentially overlooking the nuanced significance levels of individual features.

We compared these baselines on synthetic data as well as the real datasets.

## Results

### Synthetic data

**Figure 5.**
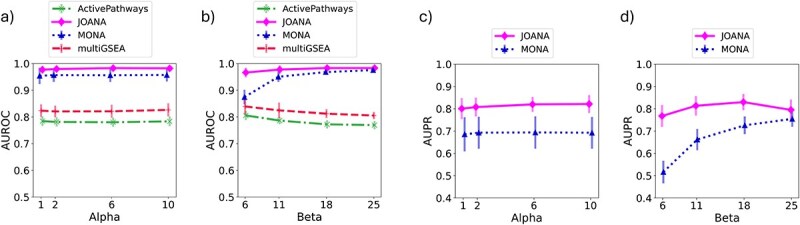
Performance of JOANA and baselines on synthetic data. AUROC on multi-omics model and its dependency on (a) Alpha and (b) Beta parameters for all of the for all baselines. AUPR for MONA and JOANA and its dependency to (c) Alpha and (d) Beta parameter.

JOANA significantly outperformed ActivePathways and multiG-SEA in terms of the average Area Under the Receiver-Operator Curve (AUROC), while demonstrating performance comparable to MONA (Fig. 5a and b, Fig. S1). A more detailed analysis using the average Area Under the curve Precision-Recall (AUPR) revealed substantial performance improvements of JOANA over MONA (Fig. 5c and d, Fig. S2). JOANA exhibited superior performance to MONA, particularly in areas where $\beta $ is smaller (i.e. when a larger area is shared between the active part and inactive part, corresponding to settings with noisy pathway annotations and/or noisy measurements with FPs/FNs in the DA). We excluded the results of ActivePathways and multiGSEA, which fell below a threshold of 0.5 for the corresponding figures (Fig. 5c and d).

In summary, our results strongly indicate that JOANA outperforms state-of-the-art baselines, particularly evident in synthetic data scenarios where the common region between active part and inactive part is larger (corresponding to larger levels of observation noise in terms of FPs and FNs). With larger value of $\mathrm{\beta }$ JOANA works similar to MONA—this in turn corresponds to a small overlap between the active and inactive part or settings with little noise in the pathway annotations and/or the DA. This highlights JOANA’s potential for revealing biologically relevant findings, particularly in difficult situations where conventional methods may struggle due to arbitrary significance assumptions, which can overlook genes that are only slightly above the set threshold and may thus be inaccurately considered as inactive.

### Real-world data

To evaluate JOANA’s performance in practical clinical and biological applications, we analyzed eight diverse multi-omics datasets spanning four distinct biological contexts: (i) integrated proteome–transcriptome measurements from two cancer studies, (ii) single-cell transcriptomic profiles across different cell types, (iii) coding and non-coding mutation driver analyses from three cancer cohorts, and (iv) a liver steatosis model with transcriptomics and epigenomics data. Collectively, these datasets demonstrate JOANA’s versatility in handling heterogeneous data types and its ability to identify biologically meaningful pathways across diverse experimental and disease-related settings.

For proteome–transcriptome integration, we analyzed lung adenocarcinoma (LUAD) histological subtypes [17] and pediatric brain tumor immune microenvironments [18]. Single-cell analyses focused on bone marrow and circulating plasma cells from multiple myeloma patients. Mutation driver analyses encompassed pan-cancer, lymphoid B-cell non-Hodgkin lymphoma (Lymph-BNHL), and skin melanoma datasets. In addition, we analyzed an integrated RNA-seq and ChIP-seq dataset derived from a high-fat and alcohol-rich diet mouse model, which is known to induce liver injury and metabolic dysfunction [23], providing a biologically well-characterized setting for validation. Below, we present detailed analyses demonstrating JOANA’s superior performance in identifying core pathway signatures while maintaining high specificity compared with existing multi-omics pathway analysis tools.

#### PT: lung adenocarcinoma

Histological heterogeneity within LUAD has important implications for treatment stratification and prognosis. We applied JOANA to proteogenomic data from Gillette *et al*. [17], who characterized molecular differences between papillary and solid LUAD subtypes. Their differential expression analysis identified 12 significantly regulated genes (FDR<0.05) after controlling for smoking status, tumor location, and driver mutations (EGFR, KRAS, STK11, TP53, and ALK fusions). Only via manual inspection, the authors of the primary publication were able to identify the distinct involvement of the TCA/Kerbs cycle in the solid subtype, and cell proliferation and survival processes in the papillary subtype [17]. In contrast, JOANA’s PEA automatically revealed 11 Gene Ontology (GO) and 7 Reactome pathways with high confidence (Table 1). Consistent with the original study’s findings, we identified two major functional clusters: TCA/Krebs cycle metabolism predominantly in solid subtypes, and cell proliferation/survival processes in papillary subtypes.

**Table 1:** Enriched pathways for LUAD on papillary and solid subtypes

Pathway	DB	Probability (%)	Related to	Literature
Condensin complex	GO	99	Cell proliferation	[32–36]
MCM complex	GO	99	Cell proliferation	[37–42]
Purine deoxyribonucleoside monophosphate	GO	99	—	
Positive regulation of neutrophil extravasation	GO	99	—	
Gamma aminobutyric acid metabolic process	GO	99	TCA/Krebs cycle	[43–47]
Positive regulation of B cell differentiation	GO	99	—	
Ethanol oxidation	GO	98	TCA/Krebs Cycle	[48, 49]
BBSOME	GO	93	—	
Branched chain amino acid catabolic process	GO	92	—	
Dihydrolipoyl dehydrogenase complex	GO	57	TCA/Krebs cycle	[50–54]
B 1 B cell differentiation	GO	53	—	
Unwinding of DNA	Reactome	99	Cell proliferation	[55–59]
Condensation of prometaphase chromosomes	Reactome	99	Cell proliferation	[60–64]
Beta oxidation of butanoyl COA to acetyl COA	Reactome	78	TCA/Krebs cycle	[65–69]
FGFR3B ligand binding and activation	Reactome	72	Cell proliferation	[70–74]
RUNX1 regulates genes involved in WNT signaling	Reactome	66	Cell proliferation	[75–79]
Ligand receptor interactions	Reactome	51	Cell proliferation	[80–84]
Beta oxidation of decanoyl COA to octanoyl COA	Reactome	51	TCA/Krebs cycle	[85–89]

Metabolic reprogramming was evident through multiple interconnected pathways. Ethanol oxidation (98% probability), which generates acetyl-CoA for TCA cycle entry, was significantly enriched alongside beta-oxidation pathways for butanoyl-CoA (78%) and decanoyl-CoA (51%). The gamma-aminobutyric acid metabolic process (99%) and dihydrolipoyl dehydrogenase complex (57%), both integral to TCA cycle function, further substantiated metabolic dysregulation in solid tumors [90].

Cell proliferation signatures dominated the papillary subtype, with DNA unwinding (99%) and prometaphase chromosome condensation (99%) representing critical mitotic processes. The MCM complex (99%), essential for DNA replication initiation, showed particularly strong enrichment—consistent with previous reports linking MCM7 expression to papillary LUAD and lymph node involvement [37, 91]. Additional proliferative pathways included FGFR3B signaling (72%), RUNX1-mediated WNT regulation (66%), and ligand–receptor interactions (51%), collectively indicating enhanced growth factor responsiveness in papillary tumors.

#### Immune microenvironment profiling distinguishes pediatric brain tumor subtypes

Tumor microenvironment composition critically influences therapeutic response and patient outcomes. We analyzed proteogenomic data from Petralia *et al*. [18], who performed consensus clustering of pediatric brain tumors based on xCell deconvolution, identifying five distinct immune clusters: Cold-medullo, Cold-mixed, Epithelial, Hot, and Neuronal.

JOANA analysis comparing the proteogenome of the immune-enriched Hot cluster against other subtypes revealed an enrichment of immune-related pathways (Table 2). Among Hallmark pathways, 83% (5/6) of significantly enriched gene sets belonged to immune categories, despite these representing only 7 pathways in the entire Hallmark collection. This underscores JOANA’s ability to capture specific and biologically coherent signatures. Additional pathway analyses based on GO [92] and Reactome [93] pathways are provided in Supplementary Data 1 and further confirm these findings.

**Table 2:** Enriched pathways for pediatric brain tumor on hot cluster and other immune clusters

Pathway	DB	Probability (%)	Related to	Literature
IL6/JAK/STAT3 signaling	Hallmark	100	Immune	[94, 98–101]
Interferon alpha response	Hallmark	100	Immune	[98, 102–104]
Interferon gamma response	Hallmark	100	Immune	[98, 105–108]
Kras signaling up	Hallmark	100	Immune	[97, 109–113]
Complement and coagulation cascades	Hallmark/KEGG	100	Immune	[95, 98, 114–116]
Allograft rejection	Hallmark/KEGG	100	Immune	[96, 98, 117]
Other glycan degradation	KEGG	100	Immune	[118–121]
Toll-like receptor signaling pathway	KEGG	100	Immune	[122–126]
Leishmania infection^a^	KEGG	100	Immune	–
Primary immunodeficiency	KEGG	100	Immune	[127–129]
FC epsilon RI signaling pathway	KEGG	99	Immune	[130–133]

^a^Leishmania is described as an intracellular protozoan parasite that primarily infects macrophages, a type of immune cell. The reason that it showed up here could be because of the common genes in immune pathways.

Key immune pathways included IL6/JAK/STAT3 signaling, interferon alpha/gamma responses, complement cascades, and allograft rejection (all 100% probability). The IL6/JAK/STAT3 axis, known to promote tumor proliferation while suppressing anti-tumor immunity through STAT3-mediated regulation of myeloid-derived suppressor cells and regulatory T cells, emerged as a central hub [94]. Complement system activation correlated with immune infiltration patterns and prognosis [95], while the allograft rejection signature strongly associated with cytolytic activity and checkpoint molecule expression across multiple immune cell types [96].

KRAS signaling upregulation (100%), though not formally classified as an immune pathway, is known for immunomodulatory effects through inflammation and immune cell recruitment [97]. Additional KEGG pathways included Toll-like receptor signaling (100%), primary immunodeficiency (100%), and Fc epsilon RI signaling (99%), collectively painting a comprehensive picture of immune activation.

**Figure 6.**
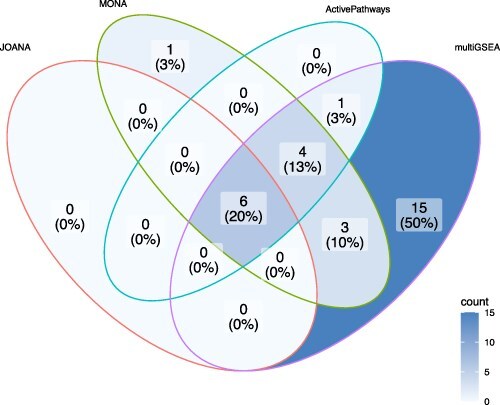
This figure illustrates the number of enriched pathways identified by MONA, ActivePathways, multiGSEA, and JOANA, as well as the number of enriched pathways that are common among these tools.

Notably, JOANA’s multi-omics integration revealed pathways that could not be recovered by single-layer analyses (i.e. proteome or transcriptome alone). While some pathways (e.g. other glycan degradation) appeared in individual omics layers, others like Fc epsilon RI signaling emerged only through integrated analysis, highlighting the importance of the integrative approach of JOANA. Comparative analysis showed that while competing methods (MONA, ActivePathways, and multiGSEA) identified the same core pathways, they reported substantially more pathways overall, demonstrating JOANA’s superior specificity (Fig. 6).

#### Single-cell transcriptomics reveals microenvironment-specific signatures in multiple myeloma

To demonstrate JOANA’s applicability to single-cell data, we analyzed transcriptomic profiles from Ledergor *et al*. [20], who compared bone marrow plasma cells (BM PCs) and circulating tumor cells (CTCs) across multiple myeloma (MM) subtypes (symptomatic and asymptomatic). Their analysis revealed that while CTCs largely mirror BM PC transcriptional states, microenvironmental differences introduce subtle but important variations.

We leveraged this biological insight by treating BM PCs and CTCs as distinct data views for multi-modal analysis characterizing differences between symptomatic MM patients and asymptomatic patients with monoclonal gammopathy of undetermined significance (MGUS). JOANA identified pathways enriched across both compartments (representing core myeloma biology) as well as compartment-specific signatures reflecting microenvironmental influences.

Core myeloma pathways enriched across both modalities included established oncogenic drivers (Table 3). Notch signaling (100%), frequently dysregulated in MM and targetable therapeutically [134], emerged alongside Hedgehog signaling (100%), which promotes MM cell survival and proliferation [135–137]. MYC target v2 (100%) highlighted metabolic vulnerabilities, as MYC overexpression sensitizes MM cells to glutamine metabolism inhibitors [138]. Peroxisome dysfunction (100%) and ribosomal activity (100%) reflected disrupted lipid metabolism and protein synthesis—key dependencies in MM cells [139, 140].

**Table 3:** Enriched pathways for single-cell MM patients on Hallmark and KEGG

Pathway	DB	Probability (%)	Literature
Notch signaling	Hallmark	100	[134, 147–150]
Hedgehog signaling	Hallmark	100	[137, 147, 151–153]
MYC target v2	Hallmark	100	[138, 154–157]
Peroxisome	Hallmark	100	[139, 158–161]
Ribosome	KEGG	100	[140]

Compartment-specific analyses revealed microenvironment-driven differences. CTCs uniquely showed cholesterol homeost -asis dysregulation, with statin treatment inducing apoptosis in MM cells [141]. Heme metabolism alterations, including HO-1 upregulation conferring bortezomib resistance [142], and coagulation abnormalities contributing to bleeding complications [143] completed the CTC-specific signature.

BM PC-specific pathways reflected the bone marrow niche influence: Wnt$\beta $-catenin signaling disruption by malignant plasma cell-secreted antagonists [144], TGF-$\beta $ signaling inhibition promoting both bone formation and MM suppression [145], and p53 pathway impairment weakening tumor cell death mechanisms [146]. These findings demonstrate JOANA’s ability to dissect microenvironment-specific biology from single-cell data through multi-modal analysis integrating single-cell data from different tissues.

#### Integration of coding and non-coding mutations reveals cancer-specific pathway vulnerabilities

Comprehensive understanding of cancer biology requires analyzing both coding and regulatory mutations. Following Zolotovskaia *et al*.’s framework [162], we applied JOANA to mutation data from the Pan-Cancer Analysis of Whole Genomes (PCAWG) project [22], treating coding region and promoter mutations as distinct modalities.

Pan-cancer analysis of significant driver mutations in coding regions and promoters using KEGG pathways demonstrated high specificity, with JOANA identifying exclusively cancer-related pathways (Table 4): colorectal, pancreatic, chronic myeloid leukemia, non-small cell lung, prostate, and endometrial cancers (99%–100% probability). This highly specific results illustrate the suitability of JOANA for mutation-based pathway analysis.

**Table 4:** Enriched pathways for coding and non-coding driver mutations Pan-cancer on KEGG

Pathway	DB	Probability (%)
Colorectal cancer	KEGG	100
Pancreatic cancer	KEGG	100
Chronic myeloid leukemia	KEGG	100
Non-small cell lung cancer	KEGG	100
Prostate cancer	KEGG	99
Endometrial cancer	KEGG	99

Lymph-BNHL analysis revealed pathways central to lymphoma biology (Table 5). The homologous recombination (HR) pathway (99%), essential for DNA repair and genomic stability, showed strong enrichment consistent with established links between HR deficiency and non-Hodgkin lymphoma risk [163–166].

**Table 5:** Enriched pathways for coding and non-coding driver mutations in Lymph-BNHL

Pathway	DB	Probability (%)	Literature
Glyceraldehyde-3-phosphate metabolic process	GO	100	[171–174]
Homologous recombination	KEGG	99	[163–165]
Taurine and hypotaurine metabolism	KEGG	73	[175]

Similarly, skin melanoma analysis uncovered both expected and novel associations (Table 6). Bladder cancer pathway enrichment (94%) reflected shared mutational signatures between melanomas and bladder cancers [167]. Estrogen-stimulated PKCz signaling (99%) aligned with emerging evidence for hormone receptor involvement in melanoma [168, 169]. The p38 MAPK pathway (67%), implicated in PMCA4b degradation and melanoma metastasis [170], represented a potential therapeutic target.

**Table 6:** Enriched pathways for coding and non-coding driver mutations in Skin-Melanoma

Pathway	DB	Probability (%)	Literature
Regulation of pentose phosphate shunt	GO	54	[176]
Bladder cancer	KEGG	94	[167]
Estrogen-stimulated signaling through PRKCZ	Reactome	99	[168, 169]
Signaling to p38 via RIT and RIN	Reactome	67	[170, 177–180]

Table 4 presents the pathways identified for Pan-cancer by JOANA using the KEGG biological dataset. The identification of all cancer-related pathways highlights JOANA’s specificity in pinpointing key pathways. It is significant that these pathways were also identified using ActivePathways and multiGSEA methodologies.

The results of JOANA on Lymph-BNHL are shown in Table 5. The HR pathway, essential for DNA repair, maintenance, and genomic stability, has gained considerable attention in cancer research due to its close association with cancer driver mutations. Defective HR can result in genomic instability, an accumulation of mutations, and ultimately contribute to tumor development. Several studies have shown that mutations affecting key components of this pathway are linked to DNA instability and an increased risk of non-Hodgkin lymphoma [163–166].

Table 6 displays enriched pathways in Skin-Melanoma. This study [170] investigates how the p38 MAPK pathway contributes to the degradation of PMCA4b, a protein known to inhibit the spread of melanoma cells. The findings suggest that targeting this pathway could offer potential treatments for slowing the progression and spread of melanoma. This research [167] also identifies a shared gene mutation in both melanoma and bladder cancer.

### JOANA identifies pathways with high specificity

**Figure 7.**
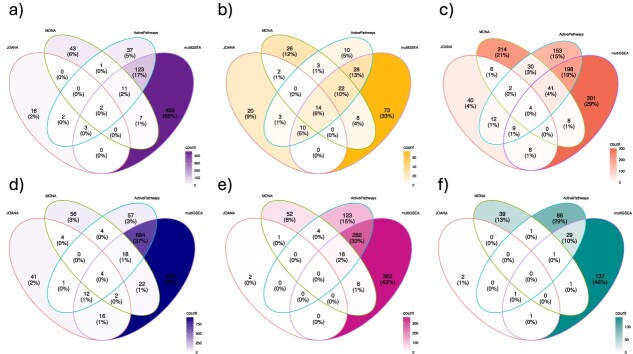
These diagrams illustrate the comparison of number of enriched pathways using real-data among JOANA, MONA, ActivePathways, and multiGSEA. (a) Results for lung-Adenocarcinoma data, (b) pediatric brain cancer, (c) single-cell multiple myeloma, (d) pan-cancer, (e) lymph-BNHL, and (f) skin-melanoma. Venn diagrams depict the number of enriched pathways identified by each tool and the pathways common between them.

To systematically evaluate specificity, we compared the number of enriched pathways identified by each method across four major databases (Hallmark, KEGG, Reactome, and GO). Figure 7 reveals a consistent pattern: JOANA reports substantially fewer enriched pathways while retaining sensitivity to biologically relevant signals.

Across all datasets, competing methods identified between 2- and 10-fold more pathways than JOANA. For example, in the pediatric brain tumor analysis (Fig. 7b), multiGSEA reported 928 enriched pathways ($\sim $50% of all tested pathways), whereas JOANA identified only 41 ($\sim $2%). Despite this difference in scale, both approaches recovered the same core immune-related signatures. This trend was consistent across LUAD (Fig. 7a), single-cell multiple myeloma (Fig. 7c), and mutation-based analyses (Fig. 7d–f).

Consistent with these observations, we evaluated the pathway enrichment method *mitch* [181], which was not included in the main benchmark due to its low precision. As shown in Fig. S11, across three real datasets *mitch* identified a substantially larger number of enriched pathways, indicative of an elevated FP rate compared with ActivePathways, multiGSEA, MONA, and JOANA.

Venn diagram analyses show limited overlap between methods beyond a small set of shared core pathways, indicating that the additional pathways reported by competing approaches predominantly reflect secondary or nonspecific signals. In contrast, JOANA’s conservative behavior arises from explicit modeling of gene dependencies and the use of continuous pathway significance scores, resulting in a focused and interpretable set of enriched pathways suitable for downstream hypothesis generation and experimental validation. Detailed pathway counts stratified by database are provided in Figs S6–S10, consistently demonstrating JOANA’s superior signal-to-noise ratio across diverse biological contexts and data types.

#### Quantitative evaluation using long-term diet-induced steatosis mouse hepatocyte data

To further demonstrate specificity in a controlled experimental context, we evaluated pathway predictions on the long-term diet-induced steatosis (LDC) mouse model [23], for which integrative transcriptomic and epigenomic analyses provide well-established biological expectations. Based on the original study, pathways related to alcohol and xenobiotic metabolism, lipid and mitochondrial metabolism, extracellular matrix (ECM) remodeling, inflammatory signaling, and growth factor-associated kinase pathways were considered biologically relevant ground truth for this model. For quantitative comparison, pathway predictions from JOANA were benchmarked against MONA and ActivePathways using pathway-level precision, recall, and F1 score. A predicted pathway was considered a true positive (TP) if it belonged to one of the biologically supported pathway categories described above. Enriched pathways outside these functional classes were treated as FPs. FNs were defined as biologically relevant pathway categories that were not recovered by a given method.

Table 7 summarizes Precision, Recall, and F1 scores based on category-level recovery rather than individual pathway counts. While all methods successfully recovered the core biological signal, JOANA achieved substantially higher precision by minimizing the number of unrelated pathway discoveries, leading to the highest overall F1 score. Competing methods exhibited higher recall at the expense of markedly increased FP rates, reflecting a tradeoff between sensitivity and specificity.

**Table 7:** Pathway-level performance comparison on the LDC mouse hepatocyte dataset

Method	TP	FP	FN	Precision	Recall	F1 score
JOANA	6	3	2	0.67	0.75	0.71
MONA	9	12	1	0.43	0.90	0.58
ActivePathways	14	38	0	0.27	1.00	0.43

#### Constraints on quantitative method comparison

We note that the quantitative evaluation was restricted to methods that operate on unsigned pathway activity scores. Rank-based enrichment methods such as multiGSEA require consistent directionality of gene-level signals to distinguish up- and down-regulated pathways. In the LDC mouse multi-omics dataset, ChIP-seq peak significance was not associated with a direction of regulatory effect, precluding a coherent integration of signed signals across modalities. Consequently, these methods were excluded from the quantitative precision–recall analysis to avoid introducing methodological bias. Nevertheless, qualitative comparisons across datasets (Fig. 7) demonstrate that JOANA recovers the same core biological signatures while reporting substantially fewer pathways, supporting its high specificity relative to rank-based approaches.

## Discussion

JOANA addresses fundamental limitations of existing methods through continuous probabilistic modeling and explicit dependency handling. In comprehensive evaluations across synthetic and real-world datasets, we demonstrate that this is consistently reflected by the identification of highly specific and biologically relevant pathways.

JOANA’s key innovation lies in its continuous modeling of q-values through Beta mixture distributions, avoiding arbitrary significance thresholds that limit traditional methods. This approach enables detection of hidden-active genes (non-significant q-values from truly active pathways) and false-active genes (significant q-values unrelated to pathway activity), providing robustness against observation noise. The Bayesian network framework naturally handles missing data and models gene-pathway dependencies, eliminating biases from imputation or data filtering.

We validated JOANA in diverse real-world datasets across different multi-modal experimental designs. In addition to proteome–transcriptome studies, we demonstrated the applicability of JOANA to single-cell data—treating cellular compartments as distinct modalities, revealing both core disease pathways and microenvironment-specific signatures in multiple myeloma. Applying JOANA to analyzing driver mutations demonstrated versatility beyond expression data.

A critical challenge in pathway enrichment is the overwhelming number of reported pathways. Our systematic comparison revealed that competing methods reported 2–10-fold more pathways than JOANA. In pediatric brain tumor analysis, multiGSEA identified 928 pathways (50% of the database) while JOANA reported only 41—yet both captured the same core biological signals. This improvement in signal-to-noise ratio addresses a fundamental limitation that makes biological interpretation of identified pathways often cumbersome.

## Conclusion

 By combining probabilistic modeling to replace threshold-based categorization with explicit dependency modeling replacing independent gene analysis, JOANA facilitates versatile and specific integration of multi-modal data. Our extensive validation demonstrates that these modeling choices translate to superior performance in identifying biologically relevant pathways while substantially reducing false discoveries.

### Limitations

Similar to many commonly used pathway analysis methods, JOANA infers pathway activity from differential abundance measurements and focuses on comparing pathway activity between conditions. To this end, pathway activity is modeled using binary latent variables. While the inferred probabilities offer a rough indication of whether pathway activation is stronger or weaker between conditions, JOANA is not designed to explicitly capture partially active or dose-dependent pathway behavior. As a result, the method is best suited for comparative analyses rather than detailed modeling of graded or continuous pathway activation levels.

A further limitation of JOANA is its increased computational runtime compared with simpler baseline methods (Fig. S3). This additional cost arises mainly from fitting the mixture of Beta distributions prior to Bayesian inference. While this step improves the quality of downstream inference, it results in longer runtimes than enrichment-based baselines such as MONA. However, explicit scalability analyses show that JOANA exhibits a sublinear increase in runtime with respect to the number of ontology terms (Fig. S4). In practice, all evaluated configurations, including large synthetic settings with $\sim $10 000 ontology terms and >30 000 features, completed in <15 min. Although JOANA is therefore slower than methods requiring only a few minutes, the additional computational cost is moderate and reflects a tradeoff for improved precision and F1 performance in identifying active terms.

An additional limitation of JOANA appears in settings where active and inactive pathways are already very clearly separated. As shown in the synthetic experiments, when there is little overlap between active and inactive signals (corresponding to larger values of the $\beta $ parameter and low noise in the data), JOANA behaves very similarly to MONA. In these cases, the extra modeling complexity of JOANA offers only limited advantages over simpler enrichment-based methods. JOANA therefore provides its greatest benefit in noisier settings, where pathway activity signals overlap substantially and are harder to distinguish.

Another limitation lies in the current implementation of JOANA in the joanapy package, which focuses on pairwise integration of omics datasets. While the underlying framework is modular and could be extended to incorporate more than two omics layers, such an extension is not implemented in the current version. We therefore consider multi-omics integration beyond pairwise settings as an important direction for future development.

**Key Points**

- JOANA is a novel pathway enrichment analysis algorithm that models gene-level significance scores as a continuous distribution, avoiding arbitrary differential analysis thresholds.
- It captures dependencies among genes and between pathways using a Bayesian network framework.
- JOANA supports multi-omics data integration and effectively handles missing values across modalities.
- Its joint modeling approach improves specificity by reducing false positives and enhancing interpretability of enriched pathways.

## Supplementary Material

Supplementary_Data_1_bbag328

Supplementary_Data_2_bbag328

Supplementary_Data_3_bbag328

Supplementary_Information2_bbag328

## Data Availability

All code is available on https://github.com/MLO-lab/joanapy. The data analyzed in this article were accessed as specified in the primary publications; no new data were generated.
