# Supplementary material for: Continuous multi-omics pathway enrichment analysis resolves hidden functional heterogeneity
Source: Brief Bioinform. 2026 Jun 29;27(3):bbag328. doi: 10.1093/bib/bbag328 (PMC13313529; doi:10.1093/bib/bbag328)
Supplement: Supplementary_Information2_bbag328 [file supplementary_information2_bbag328.pdf]

Supplementary information for

Continuous Multi-omics Pathway Enrichment Analysis Resolves Hidden Functional Heterogeneity

Table of Contents

Introduction *2*

Evaluation with synthesized data *2*

Synthesized Data Generation *2*

Component Sampling **3**

Parameter Settings **3**

Details of results on synthesized data **4**

Running time comparison **5**

Scalability of JOANA *5*

Convergeny of Expectation Propagation **6**

Results *7*

Enriched Pathway Counts Across Methods **7**

a) Lung-adenocarcinoma 7
b) Pediatric brain cancer 7
c) Pan-cancer 7
d) Lymph-BNHL 7
e) Skin-melanoma 7

Comparison of mitch with Other Pathway Enrichment Methods **10**

Top 10 Pathways Identified by JOANA in Single-Cell MGUS and MM Data **11**

MGUS: Top 10 Enriched Pathways 11
MM Top 10 Enriched Pathways 12

JOANA's output **13**

Goodness-of-fit **14**

Introduction

JOANA (Joint continuous multi-Omics enrichment ANAlysis) is a Bayesian statistical algorithm for pathway enrichment analysis (PEA) of multi-modal data. It introduces a threshold-free approach by modeling the continuous distribution of significance scores from differential expression analysis (DEA) instead of binary classification. JOANA offers the following key improvements:

1. **Accounting for Gene Significance**: Models a continuous distribution of significance scores, avoiding arbitrary thresholds.
2. **Modeling Dependencies**: Incorporates dependencies between genes within pathways and interactions between pathways.
3. **Handling Multi-Omics Data**: Integrates and analyzes data from various omics layers, capturing interdependencies.
4. **Reducing False Positives**: Employs advanced modeling techniques to yield more reliable pathway enrichment results.

JOANA was benchmarked against tools like ActivePathways, multiGSEA, and MONA, showcasing its capabilities in integrating and analyzing multi-omics data. It is implemented as the Python package **joanapy**, with an accessible GitHub repository and comprehensive tutorial to encourage reproducibility and community collaboration.

Evaluation with synthesized data

JOANA assumes that **significance scores** (derived from differential expression analysis, likely comparing gene or protein expression across conditions) can be modeled using a **mixture of three Beta distributions**. To examine how well JOANA performs on data that **does not conform** to its assumed generative model, the authors created synthetic datasets:
we generated **115,200 datasets** using **9 types of mixture models**.

Synthesized Data Generation

we generated **115,200 synthetic datasets** using various mixture models. These models were constructed by combining Beta, Exponential, Gamma, and Uniform distributions to simulate diverse data scenarios.

Component Sampling

The mixture models consisted of three types of components:

1. **Active Components**: Modeled using Beta, Exponential, and Gamma distributions.
2. **Inactive I Component**: Always modeled using a Uniform distribution.
3. **Inactive II Components**: Also modeled using Beta, Exponential, and Gamma distributions.

Parameter Settings

The parameters for the distributions were defined as follows:

- **Shape Parameters (α and β)**:
  - **α** values: {1, 2, 6, 10}
  - **β** values: {6, 11, 18, 25}

Components	Distributions		
	Beta	Exp	Gamma
Active	Beta(1, β)	Exp(β) *	Gamma(1, β) *
Inactive II	Beta(α,1)	1-Exp(α) **	1-Gamma(1, α) **
* we chose only sampled which were < 1			
** we chose only sampled which were > 0			

This approach ensured a systematic evaluation of JOANA's ability to handle diverse data, including those not directly matching its assumed generative model.

Details of results on synthesized data

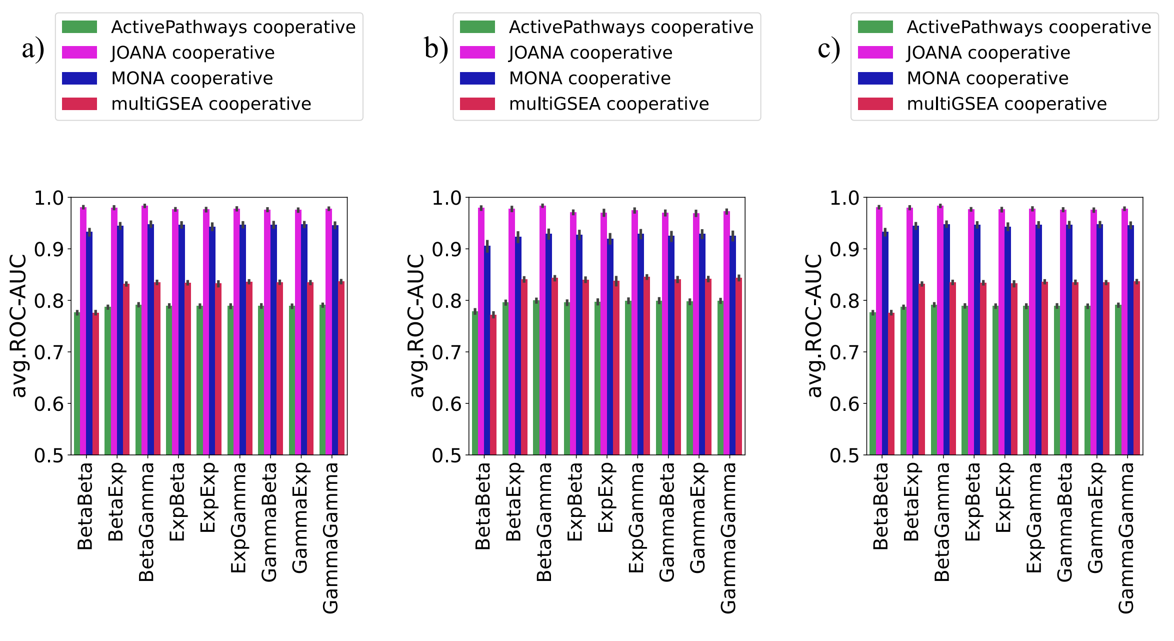

Figure S1. Comparing the average AUROC results across all mixture models and competitors, we fixed the inactive component parameter (α) while varying the active component parameter (β) under three conditions: a) β < 11, b) β < 18, and c) β < 25 for all competitors.

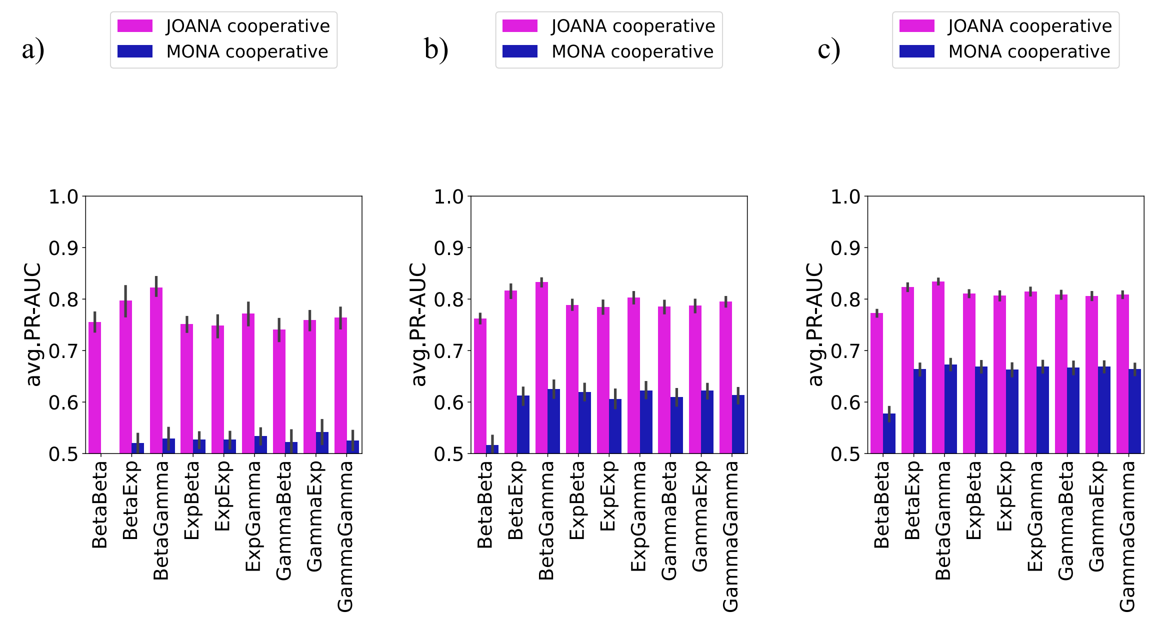

Figure S2. Comparing the average AUPR results across all mixture models and competitors, we fixed the inactive component parameter (α) while varying the active component parameter (β) under three conditions: a) β < 11, b) β < 18, and c) β < 25 for all competitors.

Running time comparison

We have also included a direct run-time comparison for all 9 synthetic data configurations (all simulated with 14,195 terms and 33632 features). While JOANA has indeed a longer run-time than baselines, all runs needed less than 15min minutes (for 10,000 ontology terms). The additional runtime in comparison to MONA stems mainly from fitting the mixture of Beta distributions. While this is longer than the 1-5min needed by baselines, it results in a substantially higher precision and F1 score in terms of identifying active terms.

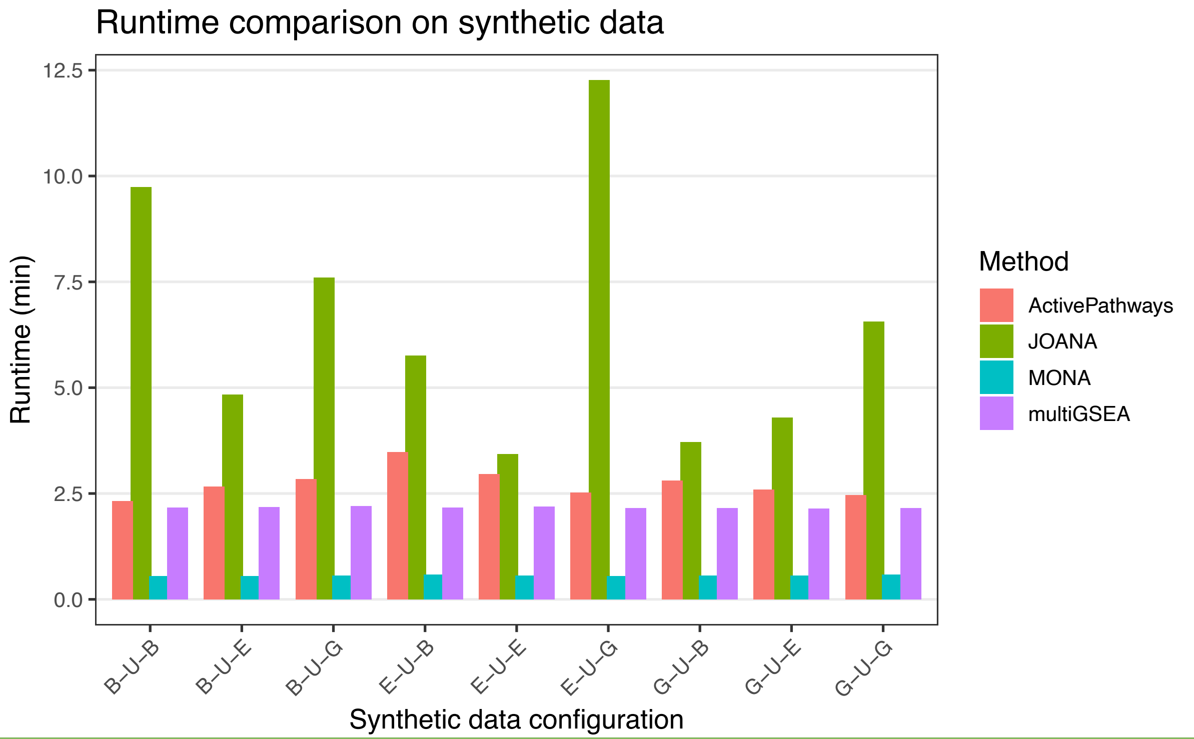

Figure S3. Runtime comparison of JOANA with MONA, ActivePathways, and multiGSEA across nine synthetic data configurations (14,195 ontology terms and 33,632 features).

Scalability of JOANA

JOANA scales with the number of ontology terms, revealing a sub-linear increase in runtime, especially for very large numbers of terms.

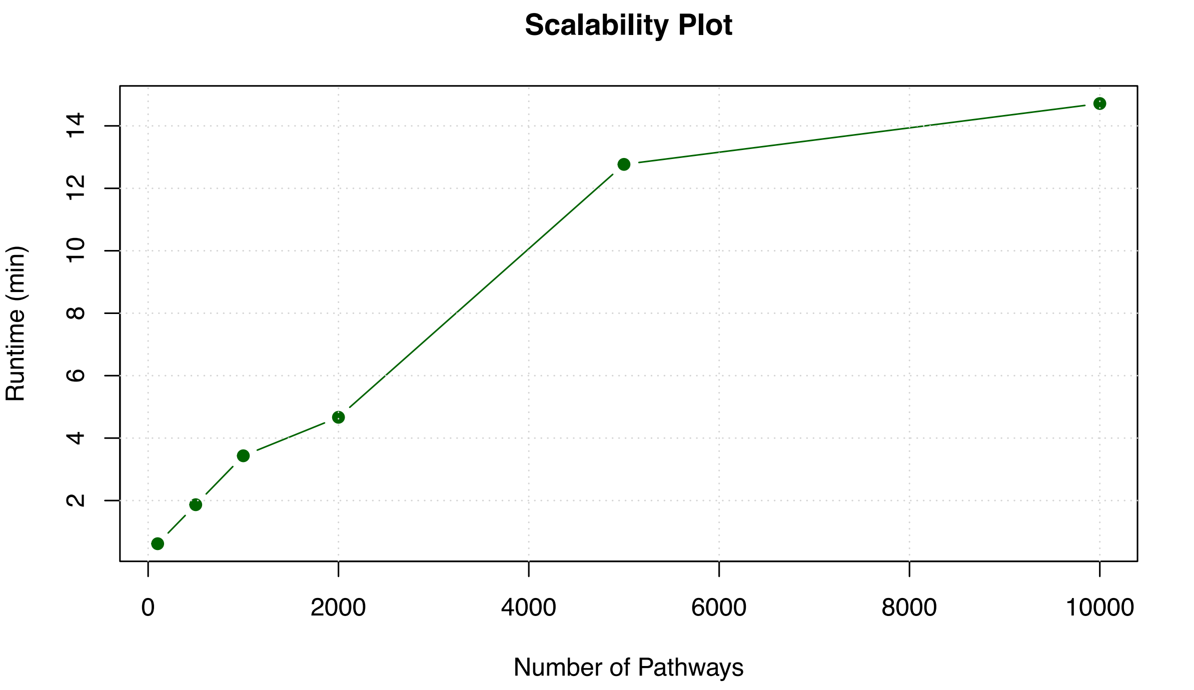

Figure S4.
Runtime of JOANA as a function of the number of ontology terms, showing a sub-linear increase in computation time as model size grows.

Convergeny of Expectation Propagation

We have performed convergence analyses by monitoring the entropy of the inferred posterior p(B|O). As expected, we observed an initially high entropy (many terms active and changing) that converged to a low value after less than 20 iterations. We run JOANA with a default of 30 iterations, ensuring convergence for all presented use-cases.

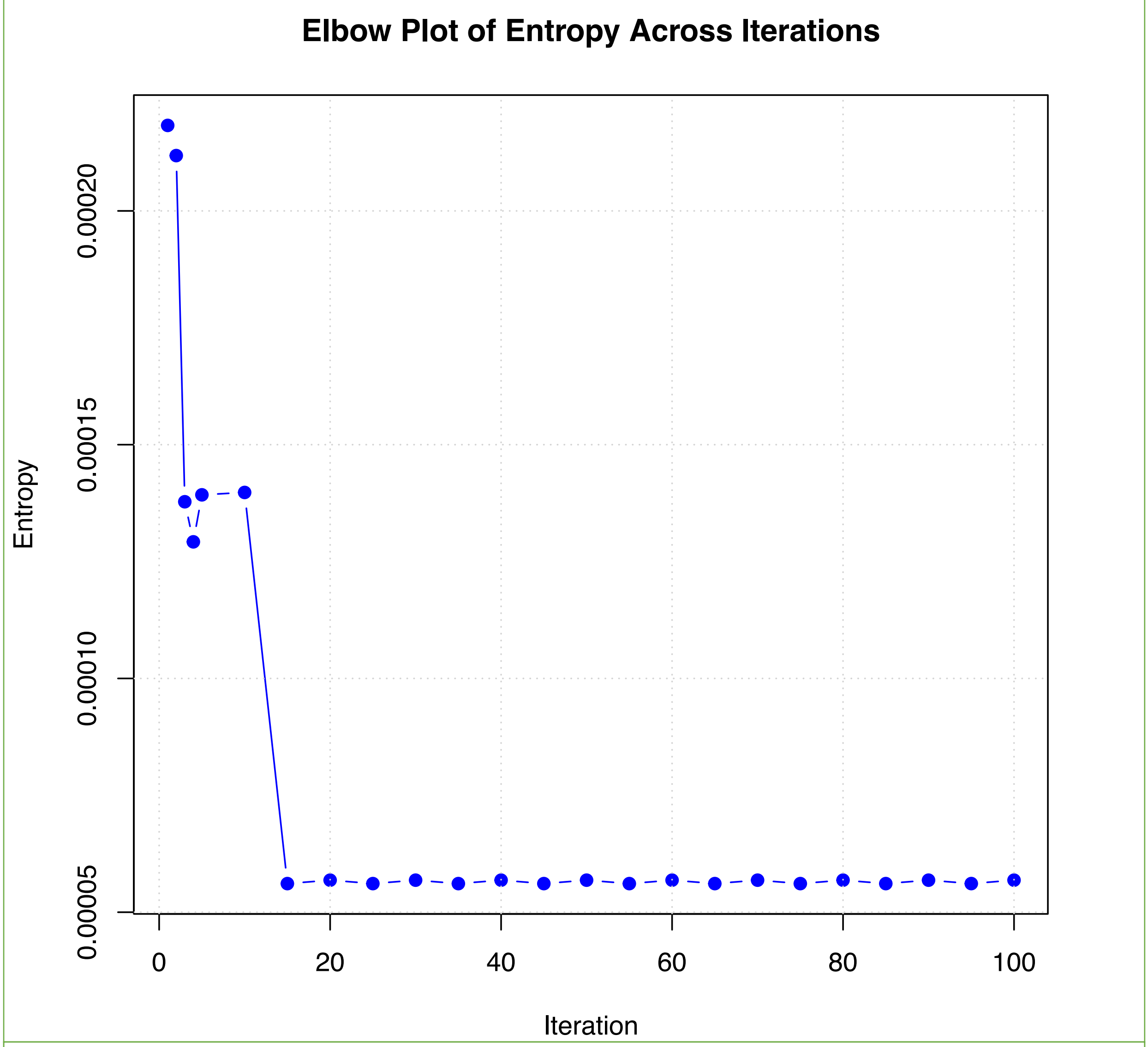

Figure S5. Entropy of the posterior p(B|O) across expectation propagation iterations. Entropy decreases rapidly and stabilizes within 20 iterations, indicating convergence. JOANA uses 30 iterations by default.

Results

Enriched Pathway Counts Across Methods

Overview of the number of enriched pathways identified by MONA, ActivePathways, multiGSEA, and JOANA across each pathway database. Results shown for:

a) Lung-adenocarcinoma data, (papillary vs Solid)
b) Pediatric brain cancer, (Hot-tumor vs other immune clusters)
c) Pan-cancer (mutated vs normal),
d) Lymph-BNHL (mutated vs normal),
e) Skin-melanoma (mutated vs normal)

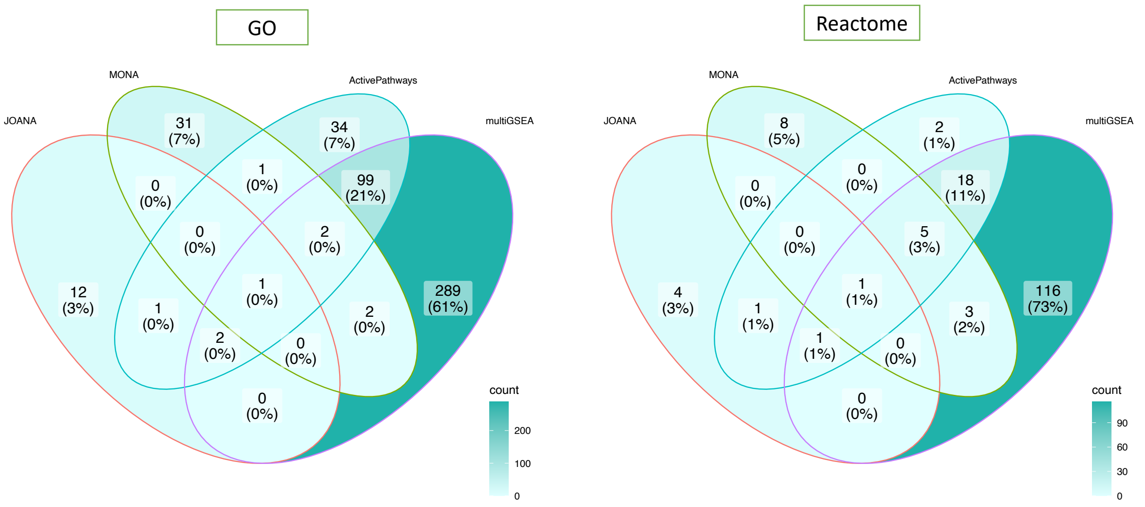

Figure S6. Pathway Enrichment in Lung Adenocarcinoma Subtypes
This figure illustrates the number of enriched pathways identified by various computational methods when comparing papillary and solid subtypes of lung adenocarcinoma. The analysis focuses on pathways from the GO and Reactome databases, highlighting differences in pathway enrichment between these histological subtypes.

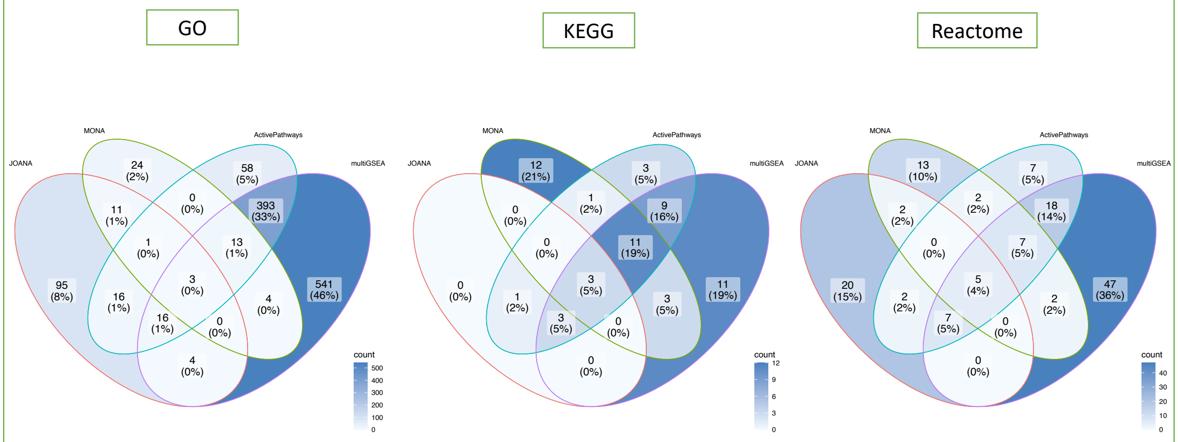

Figure S7. Pathway Enrichment in Pediatric Brain Tumor Subtypes
This figure illustrates the number of enriched pathways identified by various computational methods when comparing Hot tumor—characterized by high immune infiltration—to other tumor clusters in pediatric brain tumors. This figure shows number of pathways from the GO, KEGG, and Reactome databases, highlighting differences in pathway enrichment associated with immune infiltration levels.

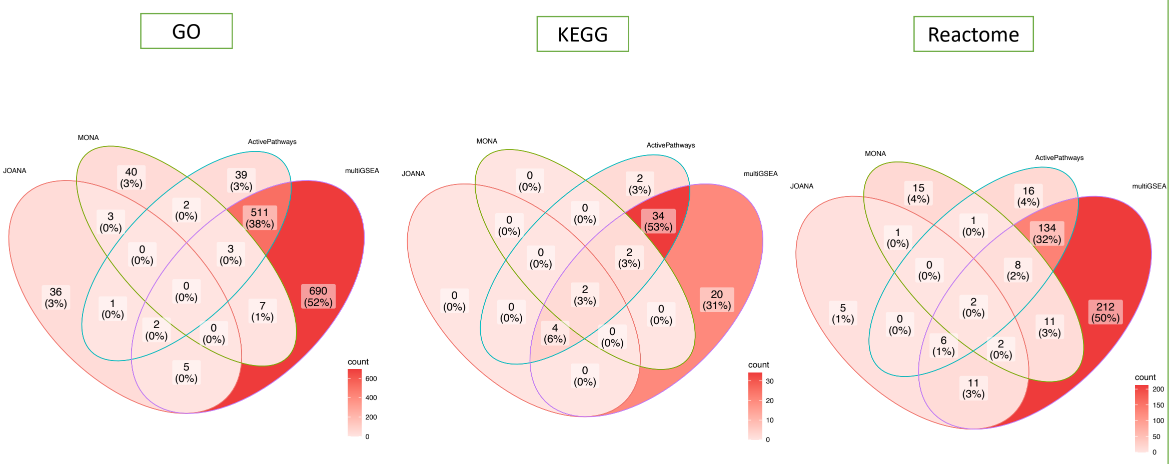

Figure S8. Pathway Enrichment for Pan-cancer on Driver Mutation
This figure illustrates the number of enriched pathways identified by various computational methods in Pan-cancer subset of the cancer driver mutation dataset (PCAWG project). The analysis encompasses multiple cancer types, highlighting the effectiveness of each method in detecting pathways associated with driver mutations.

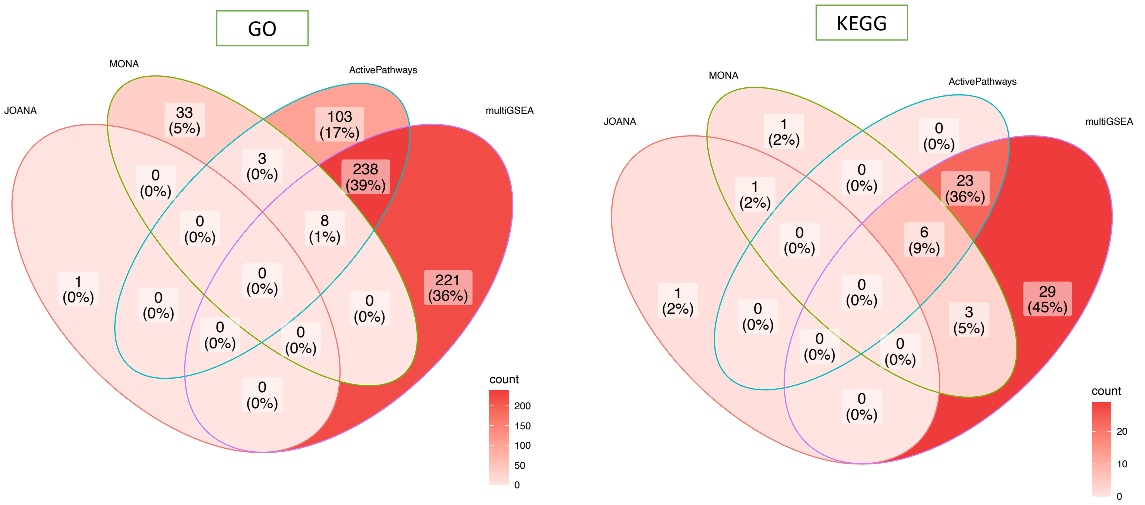

Figure S9. Pathway Enrichment in Lymph-BNHL on Driver Mutation

This figure illustrates the number of enriched pathways identified by various computational methods in the Lymph-BNHL (B-cell non-Hodgkin lymphoma) subset of the cancer driver mutation dataset (PCAWG project). The analysis highlights the comparative performance of each method in detecting pathways associated with driver mutations specific to this lymphoma subtype.

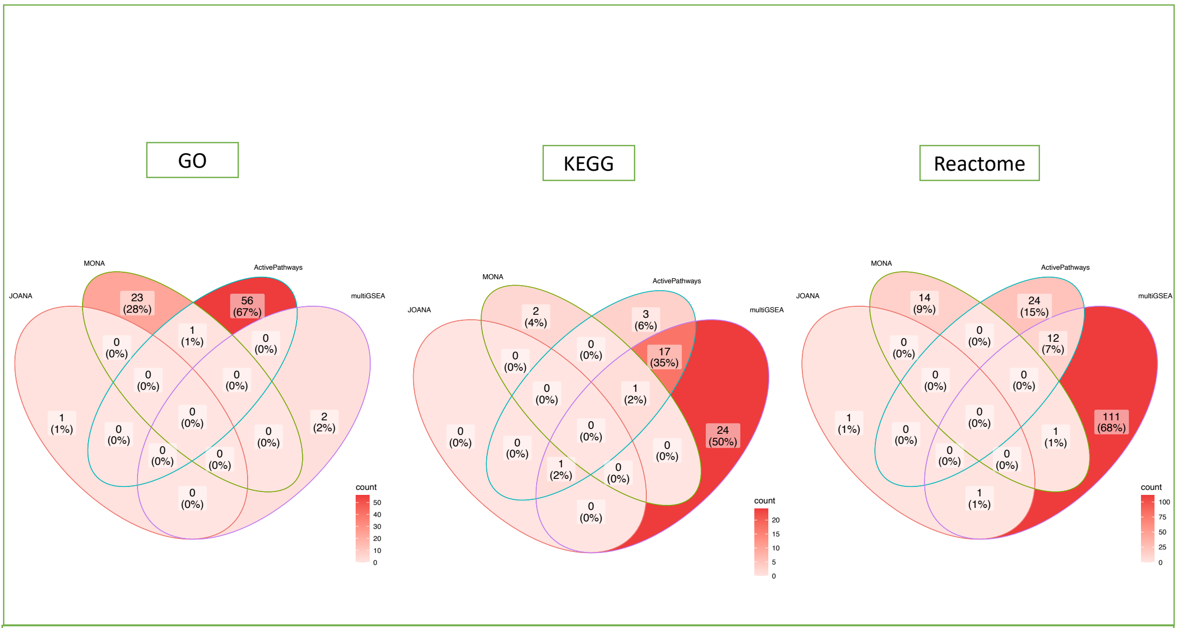

Figure S10. Pathway Enrichment in Skin-melanoma Driver Mutation

This figure illustrates the number of enriched pathways identified by various computational methods in the skin-melanoma subset of the cancer driver mutation dataset (PCAWG project). The analysis highlights the comparative performance of each method in detecting pathways associated with driver mutations specific to skin-melanoma.

Comparison of mitch with Other Pathway Enrichment Methods

We compared mitch on real world datasets and found that it tended to identify very large numbers of pathways and was not competitive with ActivePathways, multiGSEA, MONA and JOANA in terms of precision. This is illustrated in the Venn diagrams below.

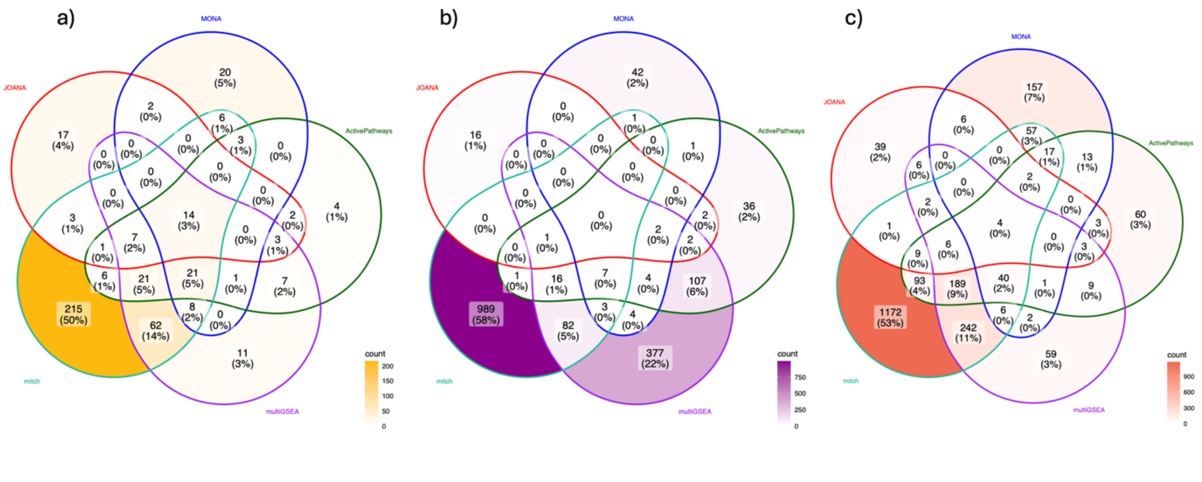

Figure S11. Venn diagrams show the overlap and number of significantly identified pathways for JOANA, ActivePathways, MONA, multiGSEA, and mitch across three representative real-world datasets. Mitch identifies a substantially larger number of pathways compared to the other methods, suggesting a broader but less selective pathway set relative to the more focused results returned by the other approaches

Top 10 Pathways Identified by JOANA in Single-Cell MGUS and MM Data

A summary of the top 10 pathways reported by JOANA for the single-cell datasets of Multiple Myeloma (MM) and Monoclonal Gammopathy of Undetermined Significance (MGUS). The complete tables are available in the Supplementary_Data_2 Supplementary_Data_3 for MM and MGUS.

MGUS: Top 10 Enriched Pathways

Table S1. Top 10 enriched pathways for MGUS on GO

Pathways	Cooperative	BM PC	CTC
GOBP_POSITIVE_REGULATION_OF_MAMMARY_GLAND_EPITHELIAL_CELL_PROLIFERATION	1.00	1.00	0.00
GOBP_POSITIVE_REGULATION_OF_VASCULAR_WOUND_HEALING	1.00	1.00	0.00000
GOBP_MITOCHONDRIAL_ELECTRON_TRANSPORT_CYTOCHROME_C_TO_OXYGEN	1.00	0.00	1.00
GOBP_COTRANSLATIONAL_PROTEIN_TARGETING_TO_MEMBRANE	1.00	0.00	1.00
GOBP_PROTON_MOTIVE_FORCE_DRIVEN_ATP_SYNTHESIS	1.00	0.00	1.00
GOBP_PROTEIN_FOLDING_IN_ENDOPLASMIC_RETICULUM	1.00	0.00	1.00
GOBP_COPI_COATED_VESICLE_BUDDING	1.00	0.00	1.00
GOBP_ENDOPLASMIC_RETICULUM_TO_CYTOSOL_TRANSPORT	1.00	0.00	1.00
GOBP_POST_TRANSLATIONAL_PROTEIN_TARGETING_TO_MEMBRANE_TRANSLOCATION	1.00	0.00	0.01
GOBP_SRP_DEPENDENT_COTRANSLATIONAL_PROTEIN_TARGETING_TO_MEMBRANE_SIGNAL_SEQUENCE_RECOGNITION	1.00	0.00	0.01

Table S2. Top 10 enriched pathways for MGUS on Reactome

Pathways	Cooperative	BM PC	CTC
REACTOME_ENDOSOMAL_VACUOLAR_PATHWAY	1.00	1.00	0.00
REACTOME_EUKARYOTIC_TRANSLATION_ELONGATION	0.00	0.00	0.99
REACTOME_ARMS_MEDIATED_ACTIVATION	0.00	0.00	0.98
REACTOME_SRP_DEPENDENT_COTRANSLATIONAL_PROTEIN_TARGETING_TO_MEMBRANE	0.00	0.00	1.00
REACTOME_P75NTR_REGULATES_AXONOGENESIS	0.00	0.00	0.99
REACTOME_NF_KB_IS_ACTIVATED_AND_SIGNALS_SURVIVAL	1.00	0.00	0.00
REACTOME_FBXW7_MUTANTS_AND_NOTCH1_IN_CANCER	0.00	0.00	1.00
REACTOME_SCAVENGING_BY_CLASS_F_RECEPTORS	0.00	0.00	1.00
REACTOME_SUMO_IS_CONJUGATED_TO_E1_UBA2_SAE1	0.00	0.00	0.68
REACTOME_LRR_FLII_INTERACTING_PROTEIN_1_LRRFIP1_ACTIVATES_TYPE_I_IFN_PRODUCTION	0.00	0.00	1.00

MM Top 10 Enriched Pathways

MM:GO

Table S3. Top 10 enriched pathways for MM on GO

Pathways	Cooperative	BM PC	CTC
GOMF_DOLICHYL_DIPHOSPHOOLIGOSACCHARIDE_PROTEIN_GLYCOTRANSFERASE_ACTIVITY	1.00	0.00	0.99
GOMF_MHC_CLASS_II_RECEPTOR_ACTIVITY	1.00	0.00	0.00
GOMF_TAP_BINDING	1.00	0.00	1.00
GOCC_MHC_CLASS_I_PEPTIDE_LOADING_COMPLEX	1.00	0.00	1.00
GOCC_SIGNAL_PEPTIDASE_COMPLEX	1.00	0.00	1.00
GOCC_ENDOPLASMIC_RETICULUM_CHAPERONE_COMPLEX	1.00	0.00	1.00
GOCC_IGG_IMMUNOGLOBULIN_COMPLEX	1.00	0.00	1.00
GOCC_SPERM_HEAD_PLASMA_MEMBRANE	0.93	0.00	0.96
GOCC_SEC61_TRANSLOCON_COMPLEX	0.73	0.00	0.66
GOCC_HRD1P_UBIQUITIN_LIGASE_ERAD_L_COMPLEX	0.48	0.00	0.54

Table S4. Top 10 enriched pathways for MM on Reactome

Pathways	Cooperative	BM PC	CTC
REACTOME_DRUG_MEDIATED_INHIBITION_OF_CDK4_CDK6_ACTIVITY	1.00	1.00	0.00
REACTOME_N_GLYCAN_TRIMMING_AND_ELONGATION_IN_THE_CIS_GOLGI	1.00	1.00	0.00
REACTOME_SRP_DEPENDENT_COTRANSLATIONAL_PROTEIN_TARGETING_TO_MEMBRANE	1.00	1.00	0.00
REACTOME_APOBEC3G_MEDIATED_RESISTANCE_TO_HIV_1_INFECTION	1.00	1.00	0.00
REACTOME_SCAVENGING_OF_HEME_FROM_PLASMA	1.00	1.00	0.00
REACTOME_UPTAKE_AND_FUNCTION_OF_DIPHTHERIA_TOXIN	1.00	1.00	0.00
REACTOME_CD22_MEDIATED_BCR_REGULATION	1.00	1.00	0.00
REACTOME_TFAP2_AP_2_FAMILY_REGULATES_TRANSCRIPTION_OF_CELL_CYCLE_FACTORS	1.00	1.00	0.00
REACTOME_HDL_CLEARANCE	1.00	1.00	0.00
REACTOME_ANTIGEN_PRESENTATION_FOLDING_ASSEMBLY_AND_PEPTIDE_LOADING_OF_CLASS_I_MHC	1.00	1.00	0.00

JOANA's output

JOANA operates in two stages:

1. **Parameterization**: In the first stage, it parameterizes the significance scores from Differential Expression Analysis (DEA) using a Beta Mixture Model (BMM).
2. **Inference**: In the second stage, it estimates the probability of pathway activity within a Bayesian Network.

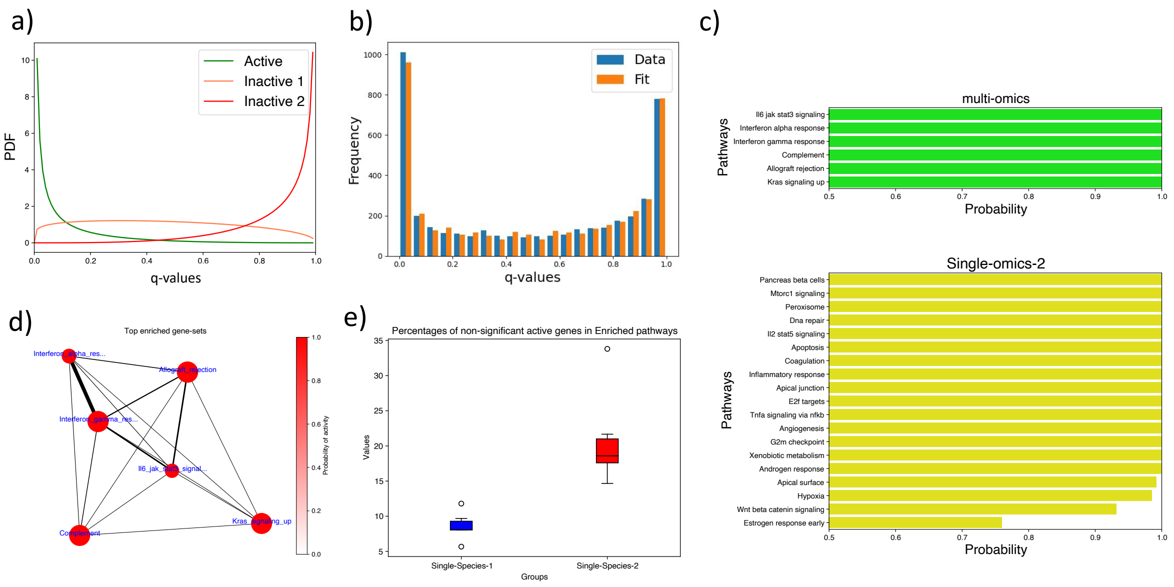

Figure S12. In this figure we showd the graphical output of joanapy.

a) Shows how the Beta Mixture Model fits the Differentialy Eexpressed Analysis data for each omics type individually.

b) it provides a barplot showing the goodness of fit for the observed data.

c) JOANA offers a PDF containing barplots that display enriched pathways with probabilities exceeding 0.5 for multi-omics (cooperative) data. It also shows pathways for each single-omics modality with probabilities >= 0.5 that do not appear in the multi-omics analysis.

d) It also includes a graph depicting the relationships between pathways. The color of the nodes indicates the probability of pathway activity, the thickness of the edges shows the degree of interconnectedness between pathways (i.e., how many common genes they share), and the size of the nodes reflects the size of the pathways.

e) Furthermore, it provides box plots and beeswarm plots that illustrate the percentage of insignificant active genes (hidden-active genes) in the enriched results.

Goodness-of-fit

As part of JOANA, we have implemented an explicit goodness-of-fit assessment that is performed after moment fitting and prior to running the Bayesian inference. This assessment compares the empirical q-value distribution obtained from differential analysis to the fitted three-component mixture model and is used to evaluate how well the modeling assumption is met for a given dataset. In Figure 2a and 2b, we show the histograms for the first and second species of one real dataset, where the empirical q-value distributions are well captured by the Beta mixture model assumed by JOANA. In Figure 2c–g, we show six additional examples with different empirical q-value distributions that clearly deviate from the mixture assumption. Even in these cases, JOANA is able to find a good match to the data, demonstrating that the method can handle a wide range of q-value distributions beyond its strict modeling assumptions.

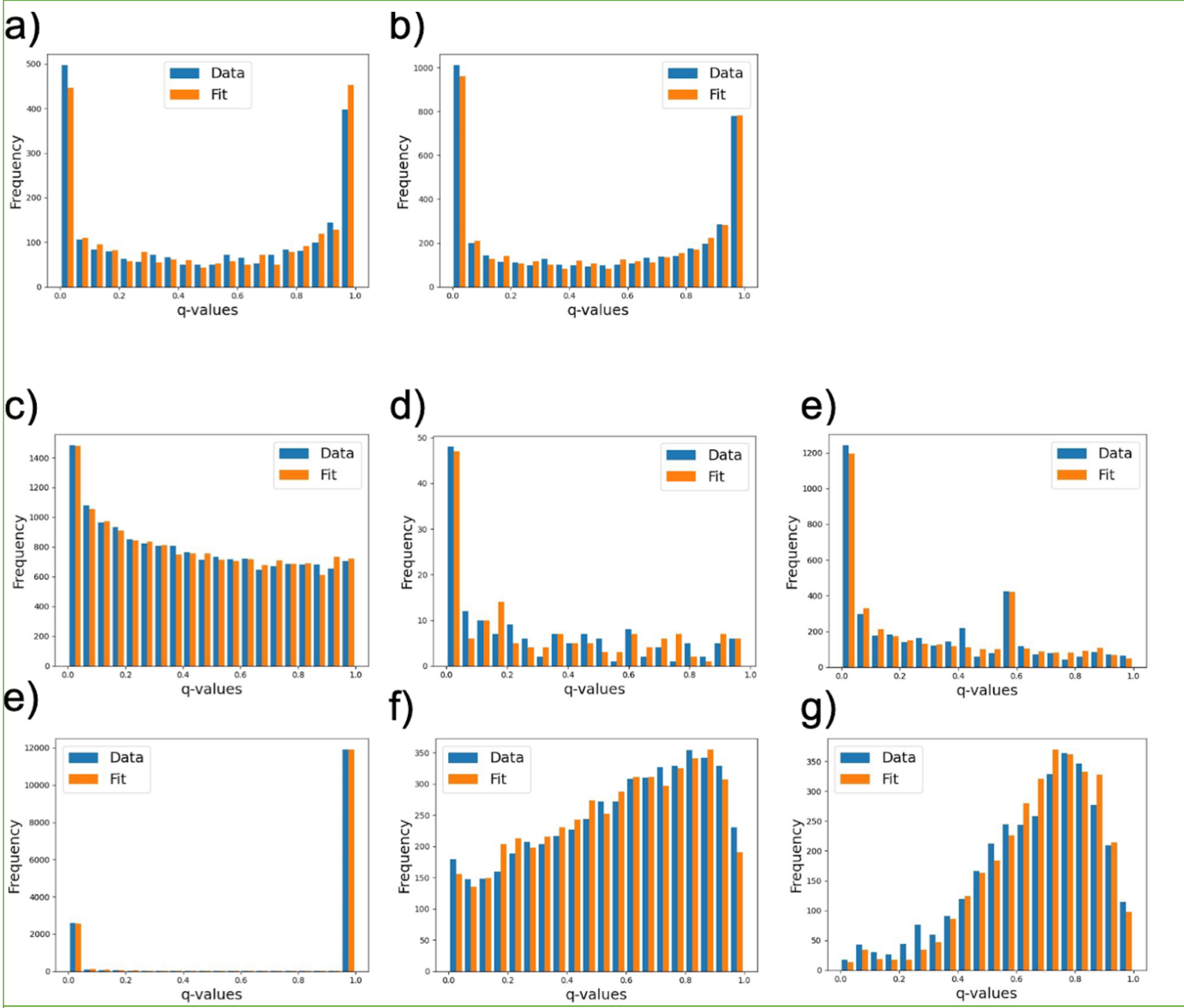

Figure S13. Goodness-of-fit assessment of JOANA on real-data q-value distributions. (a–b) Histograms of q-values for the first and second species of one real dataset, showing that the empirical distributions are well captured by the three-component Beta mixture model assumed by JOANA. (c–g) Histograms of q-values from six additional real-data examples with distributions that deviate from the mixture assumption. In all cases, JOANA is able to fit the distributions, demonstrating that the method can accommodate a wide range of empirical q-value behaviors beyond its strict modeling assumptions.
